# Exosomes derived from human amniotic mesenchymal stem cells promotes angiogenesis in hUVECs by delivering novel miRNA N-194

**DOI:** 10.1186/s10020-025-01192-8

**Published:** 2025-05-06

**Authors:** Yang Song, Tao Zhang, Ping Shi, Yingzhuo Gao, Xining Pang

**Affiliations:** 1 Department of Stem Cells and Regenerative Medicine, Shenyang Key Laboratory for Stem Cells and Regenerative Medicine, College of Basic Medicine, China Medical University, 77 Puhe Street, Shenbei New District, Shenyang City, Liaoning Province 110122 China; 2 Department of Gynecology and Obstetrics, Shengjing Hospital of China Medical University, 36 Sanhao Street, Heping District, Shenyang City, Liaoning Province 110004 China; 3 Shenyang Amnion Bioengineering and Technology R & D Center, 400-4 Zhihuier Street, Hunnan District, Shenyang City, Liaoning Province 110015 China; 4 Center of Reproductive Medicine, Shengjing Hospital of China Medical University, 36 Sanhao Street, Heping District, Shenyang City, Liaoning Province 110004 China

**Keywords:** Mesenchymal stem cells, Exosomes, MiRNAs, Angiogenesis, ING5

## Abstract

**Background:**

To investigate the effect and mechanism of exosomes derived from human amniotic mesenchymal stem cells (hAMSC-Exos) promoting angiogenesis.

**Methods:**

HAMSC-Exos were isolated using ultracentrifugation and characterized by transmission electron microscopy, NTA, and Western blot. The uptake of hAMSC-Exos by hUVECs was analyzed using PKH-26 labeling, and the effect of hAMSC-Exos on angiogenesis was analyzed in human umbilical vein endothelial cells hUVECs by cell viability assay, Transwell migration assay, Matrigel tube formation assay, and Matrigel plug assays in nude mice. Bioinformatics methods were used to analyze miRNA high-throughput sequencing data of hAMSC-Exos, and RT-qPCR was used to validate the novel miRNAs. HAMSC-Exos with high and low N-194 expression were obtained by transfection, respectively. Target genes were predicted using TargetScan, and the mRNA and protein levels of potential target genes were analyzed by RT-qPCR and Western blot after N-194 mimics transfection. Interaction between miRNAs and target genes was detected using the dual-luciferase reporter assay. Target genes were overexpressed in hUVECs by transfection. The roles of target genes in the influence of N-194 on cell function were determined by analyzing angiogenesis.

**Results:**

The extracted hAMSC-Exos showed saucer-shaped under transmission electron microscopy, and the NTA results showed the particle size of 115.6 ± 38.6 nm. The positive expression of CD9, CD63, and CD81 were verified using Western blot. The treatment of hUVECs with hAMSC-Exos significantly increased cell proliferation, migration, and angiogenesis. HAMSC-Exos contained the novel miRNAs N-194, N-314, N-19, N-393, and N-481, and the expression of N-194 was higher. The Exos derived from hAMSCs which were transfected with FAM-N-194 mimics were able to deliver FAM-N-194 mimics to hUVECs. The hAMSC-Exos with high N-194 significantly promoted angiogenesis in hUVECs. N-194 mimics transfection significantly reduced mRNA and protein levels of potential target gene ING5, and N-194 mimics significantly reduced the luciferase activities expressed by wild-type reporter gene vectors for ING5. The ING5 overexpression significantly reduced the angiogenic capacity of hUVECs. ING5 overexpression suppressed the expression of HSP27 and PLCG2.

**Conclusions:**

HAMSC-Exos promotes angiogenesis in hUVECs by delivering novel miRNA N-194 which targets ING5.

**Graphical Abstract:**

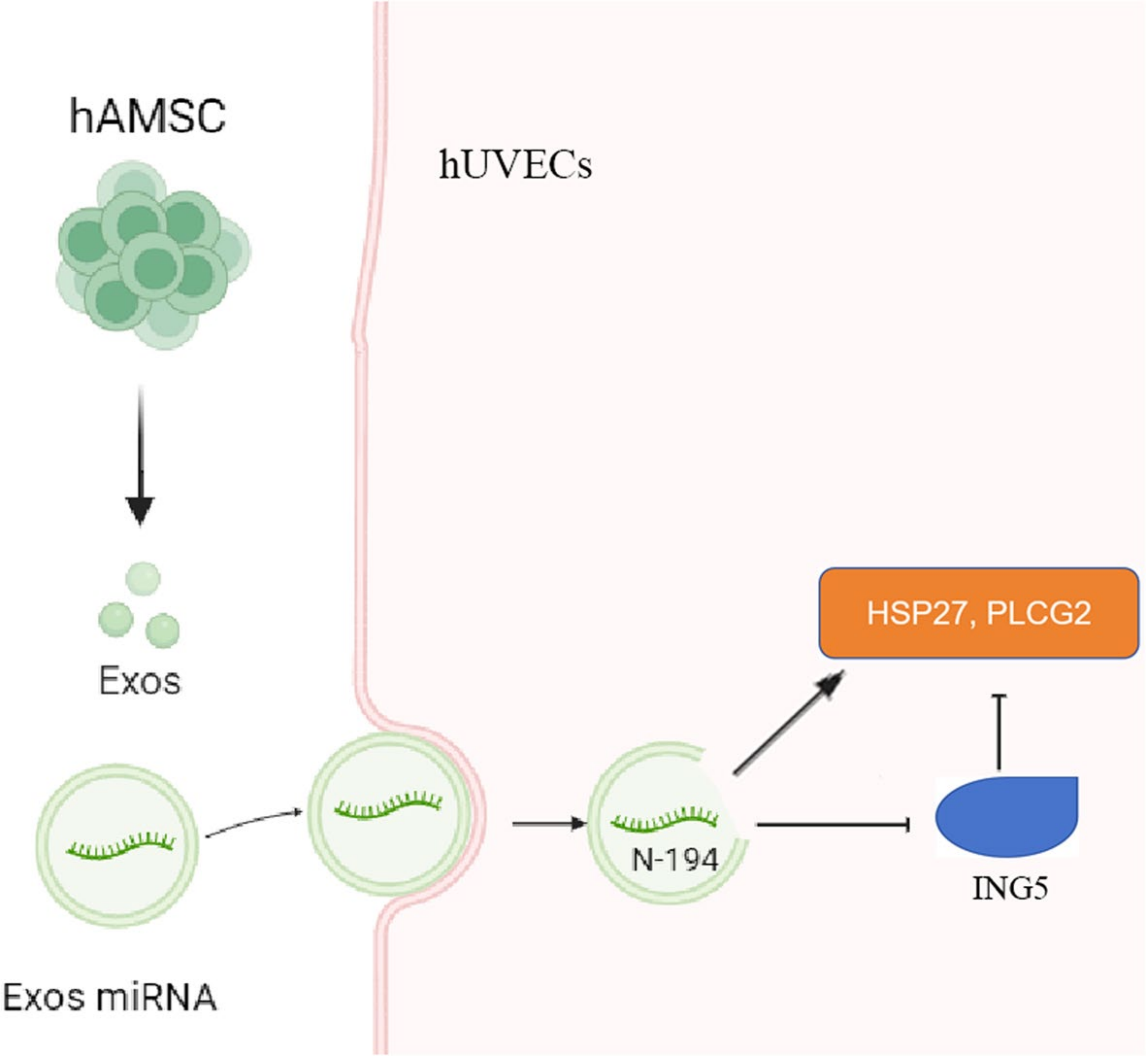

**Supplementary Information:**

The online version contains supplementary material available at 10.1186/s10020-025-01192-8.

## Introduction

The blood vessels of the human body constitute the first organ of the embryo and form the largest network in our body. It is the most important organ of the human body (Carmeliet et al. 2003). The insufficient growth of blood vessels and abnormal vascular degeneration caused by vascular abnormalities not only lead to cardiac and cerebral ischemia, but also lead to neurodegenerative diseases, hypertension, renal failure, developmental disorders, respiratory distress, organ dysfunction and other diseases (Hughes et al. 1994; Iadecola et al. 2013; Larivière et al. 2003). The repair process of many tissues including bone, heart, skin, etc. requires sufficient blood supply for the nutrition of cells (Alonzo et al. 2019; Peng et al. 2020; Roshangar et al. 2019). Vascular regeneration has become an important factor in regenerative medicine.

Mesenchymal stem cells (MSCs) are adult multipotent stem cells that originate from the mesoderm. MSCs have self-renewal and multi-directional differentiation potential and can differentiate into a variety of mesenchymal tissues (Ding et al. 2011). MSCs are distributed in the connective tissues and interstitial organs of the body. Due to their multi-directional differentiation potential and their ability to repair damaged tissues, MSC transplantation has become a new method for treating diseases (Uccelli et al. 2008). By homing effect, MSCs migrate to damaged tissues and promote tissue repair (Andrzejewska et al. 2021).

Exosomes (Exos) are disc-shaped microvesicles of approximately 30–150 nm produced by cells through exocytosis. Exos can be taken up by other cells, allowing biological signals to be transmitted between different cells (Barile et al. 2017). Exos have good storage stability and can deliver drugs and genes in vivo, which has attracted much attention in recent years (Shahabipour et al. 2017). Exos contain a variety of biological signaling molecules including microRNAs (miRNAs), mRNAs, and proteins (Toh et al. 2018; Zhang et al. 2015), which can be transmitted to target cells. Therefore, using exosomes as carriers to deliver their contents is an effective therapeutic approach (He et al. 2018). Exosomes derived from MSCs are an important mechanism for the paracrine effects of MSCs. Studies have shown that exosomes derived from MSCs can promote angiogenesis, repair damaged tissues, regulate immunity, inhibit inflammation, and suppress apoptosis (Liao et al. 2021).

Human amniotic mesenchymal stem cells (hAMSCs), a kind of embryonic derived mesenchymal stem cell, are derived from fetal amniotic membrane tissues. Human amniotic mesenchymal stem cells (hAMSCs) are one of the ideal therapeutic stem cells because of their easy accessibility, low immunogenicity, and less ethical controversy (Chen et al. 2021; Li et al. 2020), thus have the potential to be widely used in clinical research and Biomedical engineering (Farhadihosseinabadi et al. 2018). Through previous research, we found that hAMSCs have a significant promoting effect on angiogenesis by paracrine effect (Wu et al. 2017). To further clarify the role and mechanism of hAMSCs in promoting angiogenesis, we investigated the effect of exosomes derived from hAMSCs (hAMSC-Exos) on angiogenesis in this study.

## Materials & methods

### Experimental animals and cells

The male BALB/c nude mice weighing 20–25 g at 6–8 weeks of age were purchased from Beijing Huafukang Biotechnology Co., Ltd and were housed in the animal care facilities of China Medical University under specific pathogen-free (SPF) conditions. This research was approved by the Ethics Committee of China Medical University with ethics number CMU2021579. HAMSCs and human Umbilical Vein Endothelial Cells (hUVECs) were obtained from the Stem Cell and Regenerative Medicine Research Laboratory of China Medical University. HAMSCs were isolated, cultured and identified as previously described (He et al. 2020). HUVECs were cultured in RPMI 1640 medium (Gibco, USA) supplemented with 10% fetal bovine serum (FBS, Hyclone, USA) and were placed in a 37 ℃, 5% CO_2_ incubator.

### HAMSC-Exos isolation and extraction

HAMSCs of P3 generation were cultured until the confluence reached 80%, and then the medium was replaced to DMEM/F12 medium (Hyclone, USA). After cells being cultured in a CO2 incubator at 37 ℃, 5% CO_2_, the medium was collected and centrifuged at 300 g for 5 min, 2000 g for 15 min, and 13,000 g for 35 min at a time. After being filtered with 0.22 µ M sterile filter (MerckMillipore, Germany), the medium was transfered into the ultrafiltration tube (Sartorius, Germany) for ultrafiltration, and discard the lower liquid. The filter was washed with PBS, and the liquid was collected and centrifuged at 150,000 g for 3 h at 4 ℃. After the supernatant being discarded, the centrifugal sediment was dissolved in PBS and collected. The concentration of hAMSC-Exos was detected using BCA protein quantification kit (Takara, Japan).

### Transmission electron microscope

The morphology of the extracted exosomes was observed using transmission electron microscopy (Hitachi, Tokyo, Japan). 10 µL purified hAMSC-Exos was added onto the copper mesh. After 5 min, the excess liquid on the copper mesh was aspirated, and then 10 µL phosphotungstic acid was added on the copper mesh. After the copper mesh was drying, The images were displayed on 80KV-120KV.

### Nanoparticle tracking analysis (NTA)

The particle size and concentration of Exos was measured using nanoparticle tracking analysis (NTA) with ZetaView PMX 110 (Particle Metrix, Meerbusch, Germany) and software ZetaView 8.04.02. After the detection instrument was calibrating, the sample pool was washed with 1×PBS buffer. After that, the sample was diluted with 1×PBS buffer, and detected.

### Western blot

The total protein extraction kit (Takara) was used for protein extraction. BCA assay kit (Takara) was used to determine the concentration of extracted protein.

30 µg protein sample was separated by SDS polyacrylamide gel, and transferred to PVDF membrane (MerckMillipore). The PVDF membrane was incubated in 5% nonfat milk powder blocking solution for 2 h at room temperature. The PVDF membrane was cut according to the molecular weight of the protein, placed in diluted primary antibody solution (CD9 antibody 1:1000, CD63 antibody 1:1000, CD81 antibody 1:1000, Boster, USA) at 4 ℃ overnight. Horseradish peroxidase-labeled secondary antibody diluted in 5% BSA (1:10000) was added and incubated for 1 h at room temperature. The PVDF membrane was detected on a Tanon-5200 chemiluminescence detection system (Tanon, Shanghai, China) using ECL kit (Solarbio, China).

### Cell viability assay

HUVECs were routinely cultured in hUVECs complete medium (RPMI 1640 medium + 10% FBS). 5 × 10^3^ cells with 200 µL hUVECs complete medium in each well of a 96 well plate were cultured at 37 ℃ in a 5% CO2 incubator for 24, 48, 72 and 96 h, respectively. Each well was added with a final concentration of 0, 50, and 100 µg/mL of hAMSC-Exos. At each time point, each well was added with 20 µL MTS (Promega, USA) and incubated in the incubator for 2 h. The OD value of each well at 492 nm wavelength was detected.

### Transwell migration assay

To detect the migration ability of hUVECs, 5 × 10^4^ cells mixed with 100 µL RPMI 1640 medium were placed in the upper chamber of the Transwell chamber (Corning, USA), and RPMI 1640 medium supplemented with 10% FBS was added into the lower chamber. 0, 50 and 100 µg/mL of hAMSC-Exos were added to the upper chamber of the Transwell chamber, respectively. After 12 h of incubation at 37 ℃ with 5% CO2, the Transwell chamber was removed, and the chamber membrane was removed and stained with hematoxylin and eosin (HE) staining kit (Beyotime, China). Cell statistics were performed on the lower surface of the Transwell chamber membrane. The number of cells that had migrated to the lower surface of the membrane was quantified by counting 4 independent symmetrical visual fields under the microscope.

### Matrigel tube formation assay

200 µL Matrigel (354262, BD Biosciences, USA) was spread on a 24 well plate and incubated at 37 ℃ for 1 h to solidify. 5 × 10^4^ hUVECs mixed with 200 µL hUVECs complete medium were placed on the upper layer of Matrigel. 0,50 and 100 µg/mL of hAMSC-Exos were added to the medium. After 12 h of incubation at 37 ℃ with 5% CO2, the number of tubes was quantified by counting 4 independent symmetrical visual fields under a inverted microscope. The branching points from each sample were examined with Image-Pro Plus software 6.0.

### Matrigel plug assays in nude mice

RPMI 1640 medium and 250 µL Matrigel was mixed at a ratio of 1:1 at 4 ℃. 0, 50 and 100 µg/mL of hAMSC-Exos were added. The male BALB/c nude mice weighing 20–25 g were injected with a total of 500 µL of the mixture subcutaneously in the dorsal region. The plug was restored after 2 weeks. Tissue sections, HE staining and Immunohistochemistry were further used to detect the angiogenesis in vivo.

### Bioinformatics analysis

MiRDeep 2.0 was used to analyze candidate new miRNAs in previous next-generation small RNA sequencing data. RNAfold (http://rna.tbi.univie.ac.at/cgi-bin/RNAWebSuite/RNAfold.cgi) was used to analyze the secondary structure, free energy, gene locus and other information of candidate new miRNAs.

### RT-qPCR

RNAiso (Takara) was used to isolate the total RNA of cells and hAMSC-Exos. CDNA was synthesized using a Mir-X miRNA First-Strand Synthesis Kit or PrimeScript™ RT reagent Kit with gDNA Eraser kit (Takara) with normal primers or stem loop RT-qPCR primers. The synthesized cDNA was diluted into cDNA working solution by adding 4 volumes of RNase water (Takara). TB Green™ Premix Ex Taq™ II kit (Takara) was used to detect the expression of mRNAs and miRNAs. The relative levels of genes were calculated by 2–ΔΔCT method, with GAPDH or U6 as the internal reference. Primers were shown as Supplementary Tables 1 and 2.

### Cell transfection

HAMSCs were cultured until the confluence reached 50–60%, and the complete medium of hAMSCs was replaced with serum-free DMEM/F12 medium. Lipo2000 transfection reagent (Thermofisher, USA) was used to transfect the new miRNAs N-194 (N-194) mimics (GenePharma, China), short hairpin (sh)-N-194 (GenePharma) and their corresponding negative control (NC, GenePharma) into hAMSCs. After 6 h, the medium was changed to complete medium of hAMSCs. HAMSC-Exos or hAMSCs were collected after 24 h for further experiments.

### Target gene prediction

It was found that N-194 had the same seed sequence as hsa-miR-4467. TargetScan human 8.0 was applied to predict the target genes of hsa-miR-4467. The binding sites of potential target genes of hsa-mir-4467 and N-194 were analyzed.

### Dual luciferase reporter assay

Pyrobest™ DNA polymerase PCR kit (Takara) was used to amplify the wild-type DNA sequence around the predicted binding sites of ING5. The mutant DNA sequence around the binding site was synthesized. The amplified wild-type and mutant DNA sequences and pGL3 control vector were digested by Xba I restriction enzyme (Takara), and combined the amplified DNA sequences with pGL3-control vector to construct the corresponding wild-type and mutant firefly luciferase reporter vectors, respectively. Cells were cultured in 24 well plates until the confluence reached 60–70% and serum-free medium was replaced. The experiment was divided into experimental group and control group. In the experimental group, lipo2000 transfection reagent was used to transfect 2 µg firefly luciferase reporter, 0.2 µg Renilla luciferase reporter and 2 µL of N-194 mimics were cotransfected into cells. In the control group, lipo2000 transfection reagent was used to transfect 2 µg firefly luciferase reporter, 0.2 µg Renilla luciferase reporter and 2 µL of NC into cells. After 6 h, the medium was changed to complete medium with FBS. After 24 h, the cells were collected and analyzed by dual luciferase reporter assay system (Promega). Renilla luciferase was used as an internal reference to calculate the relative expression of firefly luciferase. Primers and DNA sequences were shown as Supplementary Table 3.

### Statistical analysis

Each experiment was repeated for at least three times. GraphPad. Prism v5.0 and Image J were used to analyze data and image results. The experimental results were expressed as mean (standard deviation). T-test was used for statistical analysis of the two samples, and *p* < 0.05 was considered as statistically significantly different.

## Results

### Identification of hAMSC-Exos

**Fig. 1:**
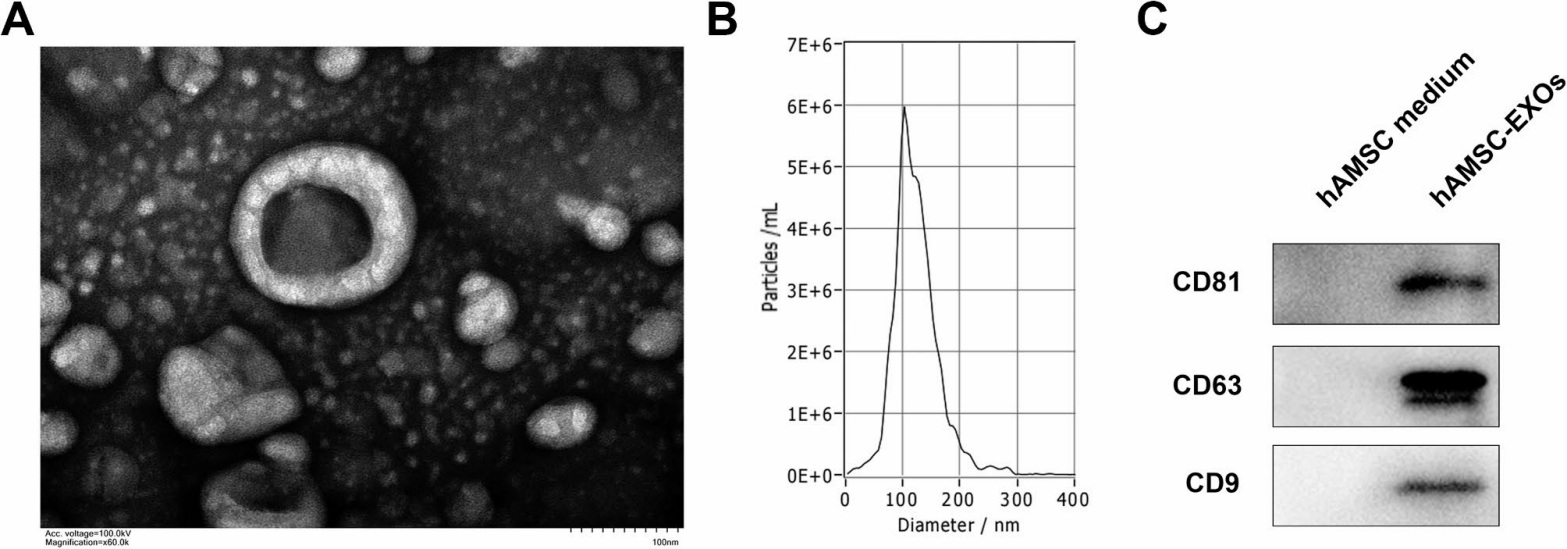
Identification of hAMSC-Exos (**A**) The morphology of hAMSC-Exos was observed using transmission electron microscopy. Scale bar = 100 nm. (**B**) The hAMSC-Exos particle size distribution was detected using NTA. (**C**) Western blot was used to detect the expression of hAMSC-Exos surface proteins CD9, CD63 and CD81

HAMSC-Exos were isolated by ultrafiltration combined with ultracentrifugation, and identified by transmission electron microscopy, NTA and Western blot. As shown in Fig. 1A and B, hAMSC-Exos showed saucer-shaped under transmission electron microscope, and NTA detection results showed that 95% of the measured sample diameter was concentrated at 115.6 (38.6) nm, which was consistent with the size of exos. Western blot showed that hAMSC-Exos highly expressed exosome surface specific markers CD9, CD63 and CD81 (Fig. 1C). These demonstrated the features of the isolated hAMSC-Exos consistent with the characteristics of Exos.

### HAMSC-Exos promote the viability, migration and angiogenesis of hUVECs

**Fig. 2:**
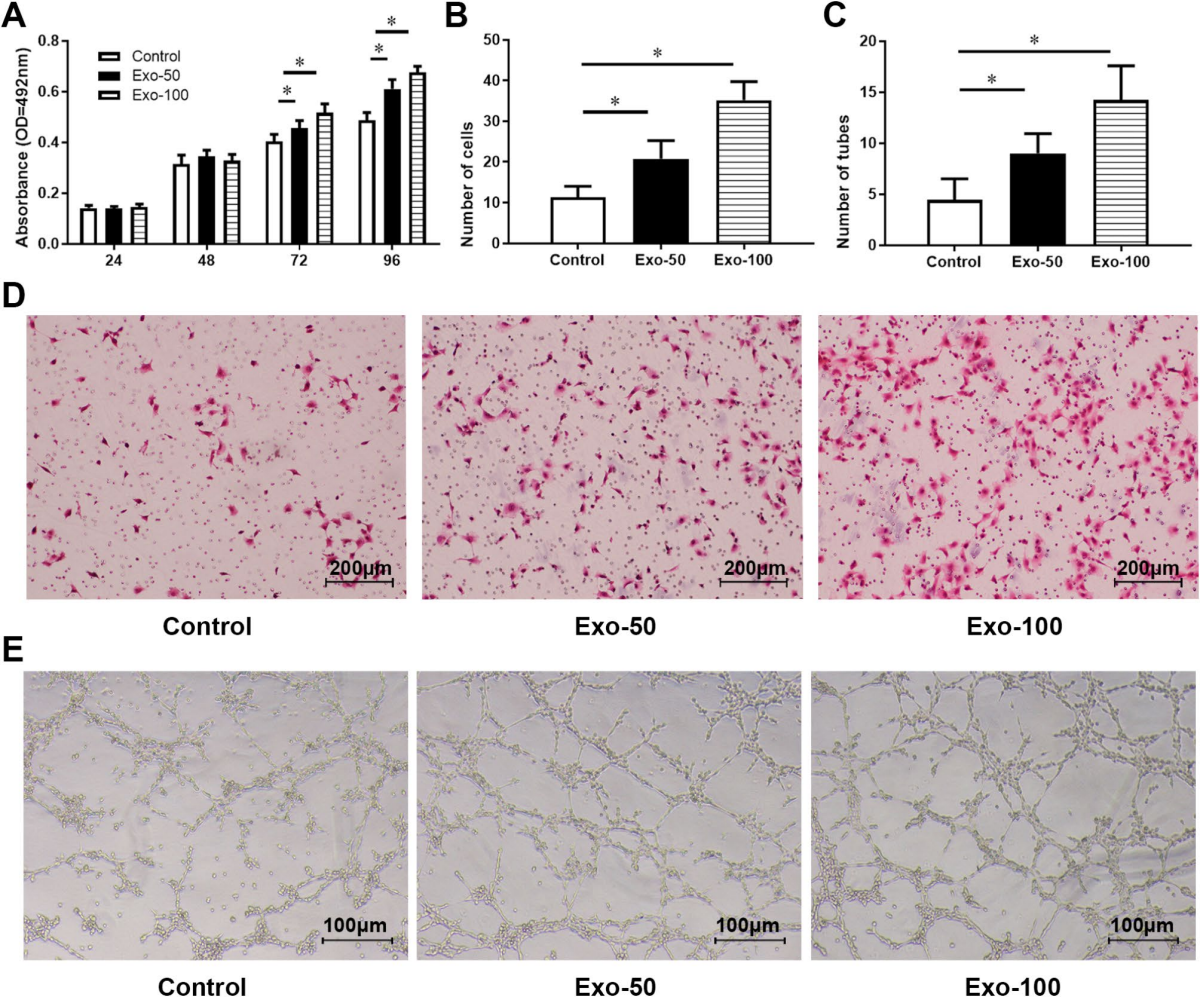
Effect of hAMSC-Exos on viability, migration and angiogenesis of hUVECs (**A**) Cell viability assay was used to detect the viability of hUVECs after being co-cultured with 50 and 100 µg/mL of hAMSC-Exos, *n* = 6. (**B**) Transwell was used to detect the effect of 50 and 100 µg/mL of hAMSC-Exos on cell migration, *n* = 12. (**C**) Matrigel tube formation assay was used to detect the effect of 50 and 100 µg/mL of hAMSC-Exos on cell angiogenesis in vitro, *n* = 9. (**D**) Representative photographs of Transwell migration assay. Scale bar = 200 μm. (**E**) Representative photographs of Matrigel tube formation assay. Scale bar = 100 μm. Data were presented as mean (standard deviation). * *P* < 0.05, compared with the control group

**To investigate the effect of hAMSC-Exos on the viability of hUVECs**, 50 µg/mL and 100 µg/mL hAMSC-Exos were added to the medium of hUVECs and cultured in the incubator. Cell viability assay was used to detect the viability of cells. It was found that 50 and 100 µg/mL of hAMSC-Exos significantly promoted the viability of hUVECs (Fig. 2A). To further detect the migration ability of hUVECs, Transwell migration assay was used. It was found that 50 and 100 µg/mL of hAMSC-Exos significantly promoted the migration ability of hUVECs (Fig. 2B and D). Matrigel tube formation assay further demonstrated that 50 and 100 µg/mL of hAMSC-Exos significantly promoted the angiogenesis of hUVECs (Fig. 2C and E). These results demonstrated that hAMSC-Exos promote the viability, migration and angiogenesis of hUVECs.

### HAMSC-Exos promote angiogenesis in nude mice

**Fig. 3:**
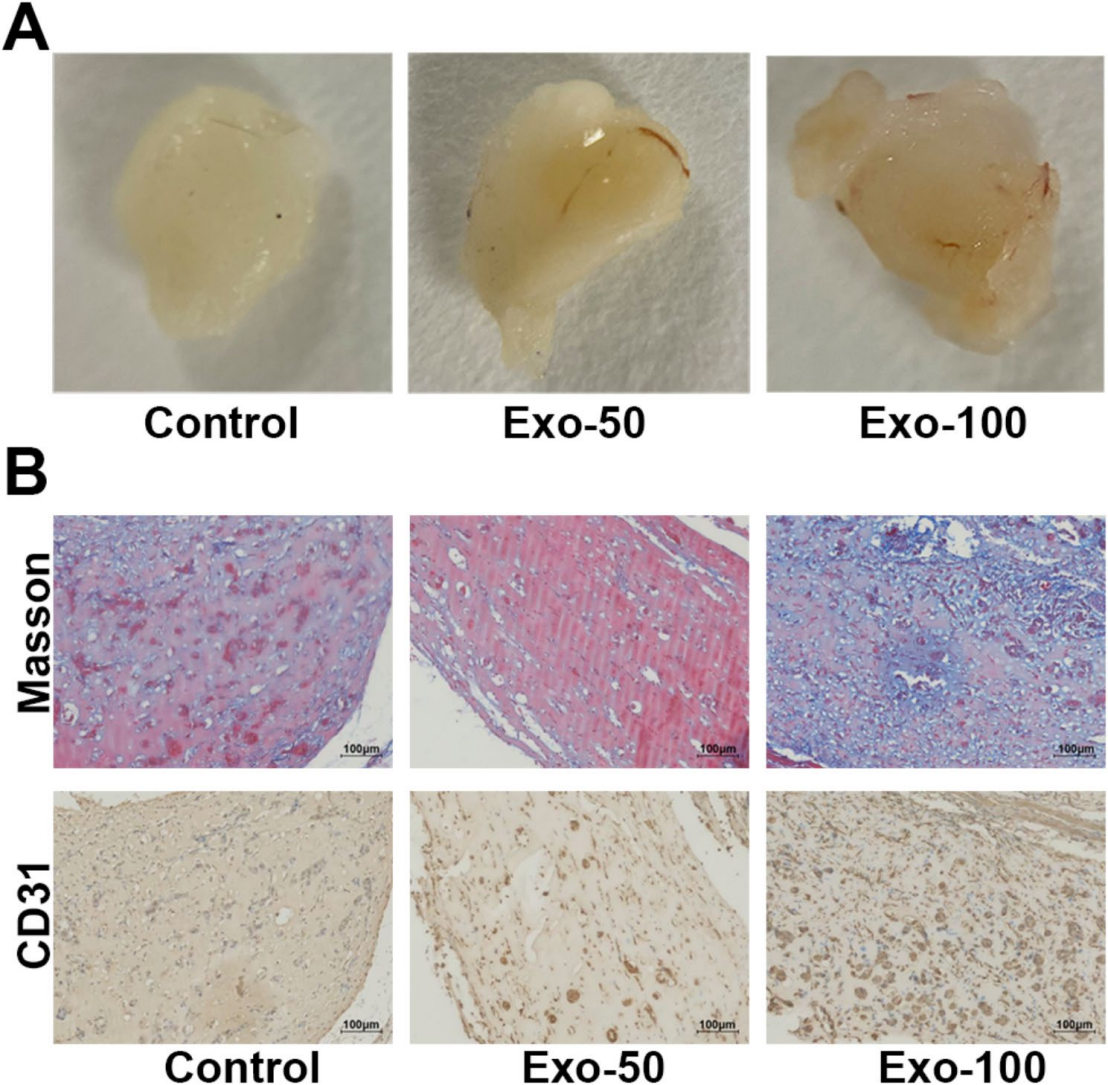
Effect of hAMSC-Exos on angiogenesis in nude mice. (**A**) The effect of hAMSC-Exos on angiogenesis in vivo was detected using Matrigel plug assays in nude mice. The results showed that 50 µg and 100 µg hAMSC-Exos significantly promoted angiogenesis in nude mice. (**B**) Masson staining and IHC staining of CD31 revealed that 50 µg and 100 µg hAMSC-Exos promoted angiogenesis in nude mice. Scale bar = 100 μm

To further investigate the effect of hAMSC-Exos on hUVECs, hAMSC-Exos or RPMI 1640 medium were mixed with Matrigel and transplanted subcutaneously into nude mice for in vivo tube forming experiments. 250 µL RPMI 1640 medium containing 50 µg hAMSC-Exos, 250 µL RPMI 1640 medium containing 100 µg hAMSC-Exos or 250 µL RPMI 1640 medium was mixed with 250 µL of Matrigel and inoculated subcutaneously in the dorsal region of mice. Two weeks later, Matrigel was removed. The angiogenesis ability was detected by Masson staining and Immunohistochemistry. It was found that 50 µg and 100 µg of hAMSC-Exos significantly promoted the expression of collagen and CD31 which is significantly related to angiogenesis (Fig. 3).

### HAMSC-Exos express a new MiRNA N-194

To further investigate the mechanism of hAMSC-Exos promote angiogenesis, next-generation small RNA sequencing of hAMSC-Exos were analyzed. New miRNAs N-194, N-314, N-19, N-393 and N-481 were predicted by miRDeep 2.0, and RNAfold was used to analyze the secondary structure and minimum free energy of the new miRNAs (Supplementary Fig. 1 and Table 1). The expression of N-194 was very high in the hAMSC-Exos according to the Heatmap (Supplementary Fig. 2). Stem loop RT-qPCR was used to detect the expression levels of new miRNAs in hAMSC-Exos, it was found that the ∆CT value of N-194 was the lowest, indicating that its expression was higher than that of other new miRNAs (Supplementary Fig. 3).

**Table 1:** …

novel miRNAs	sequences	pre-miRNA	minimum free energy	UCSC LOCATION	seed sequence conservation	Raw Counts			Normalization Couts			Average
						A1	A2	A3	A1	A2	A3	
chr15_194	CGGCGGCGGCGGCGGCGGCGGG	CGGCGGCGGCGGCGGCGGCGGGGGAAGGAUGCAGGGGAAGAAGCCGGGCGGUUCGUCGGGCGGCGGCCGGAGCGGCGAGCU	-34.50 kcal/mol	chr15:91853881-91853961	hsa-miR-4467	109	391	507	3814.79	14543.43	9055.19	9137.80
chr18_314	CGGCGGUGGCGGCGGCGGCGGCG	CGGCGGUGGCGGCGGCGGCGGCGGGGGCGGCUGCGGCAGGGGGGCCGGGGCCGCCGCCAGGGCCAGGGCCGCGGAGCUGCCGCU	-61.30 kcal/mol	chr18:51197231-51197314		11	15	13	384.98	557.93	232.18	391.70
chr10_19	CGGCGGCGGCGGCGGCGGCGGG	CGCCGCCGCCGCCGCCGCCGCAGCAGCGGCGGCCUACAGCAGCAGCUAUGGCUGUGCGUACCCGGCGGGCGGCGGCGGCGGCGGCGGCGGG	-74.20 kcal/mol	chr10:99535451-99535541	hsa-miR-4467	109	391	507	3814.79	14543.43	9055.19	9137.80
chr1_393	UCAAAUCCUGUCUGACCCU	GUGUUUCAGACAGACUGUUGAGGGAGAAGUUUUUAAGCAGAAGACUUCCCAUGUCUCUCAAAUCCUGUCUGACCCU	-28.50 kcal/mol	chr1:62101716-62101791		0	1	0	0	37.2	0	12.40
chr20_481	CGGCGGCGGCGGCGGCGGUG	CCGCCGCUGCUGCUGCUGCUGCUGCCCUGUGAGGCCGAGGCCGCGGCGGCGGCGGCGGCGGUG	-50.00 kcal/mol	chr20:3471315-3471377	hsa-miR-4467	5	14	51	174.99	520.74	910.88	535.54

### HAMSC-Exos deliver transfected N-194 to hUVECs

**Fig. 4:**
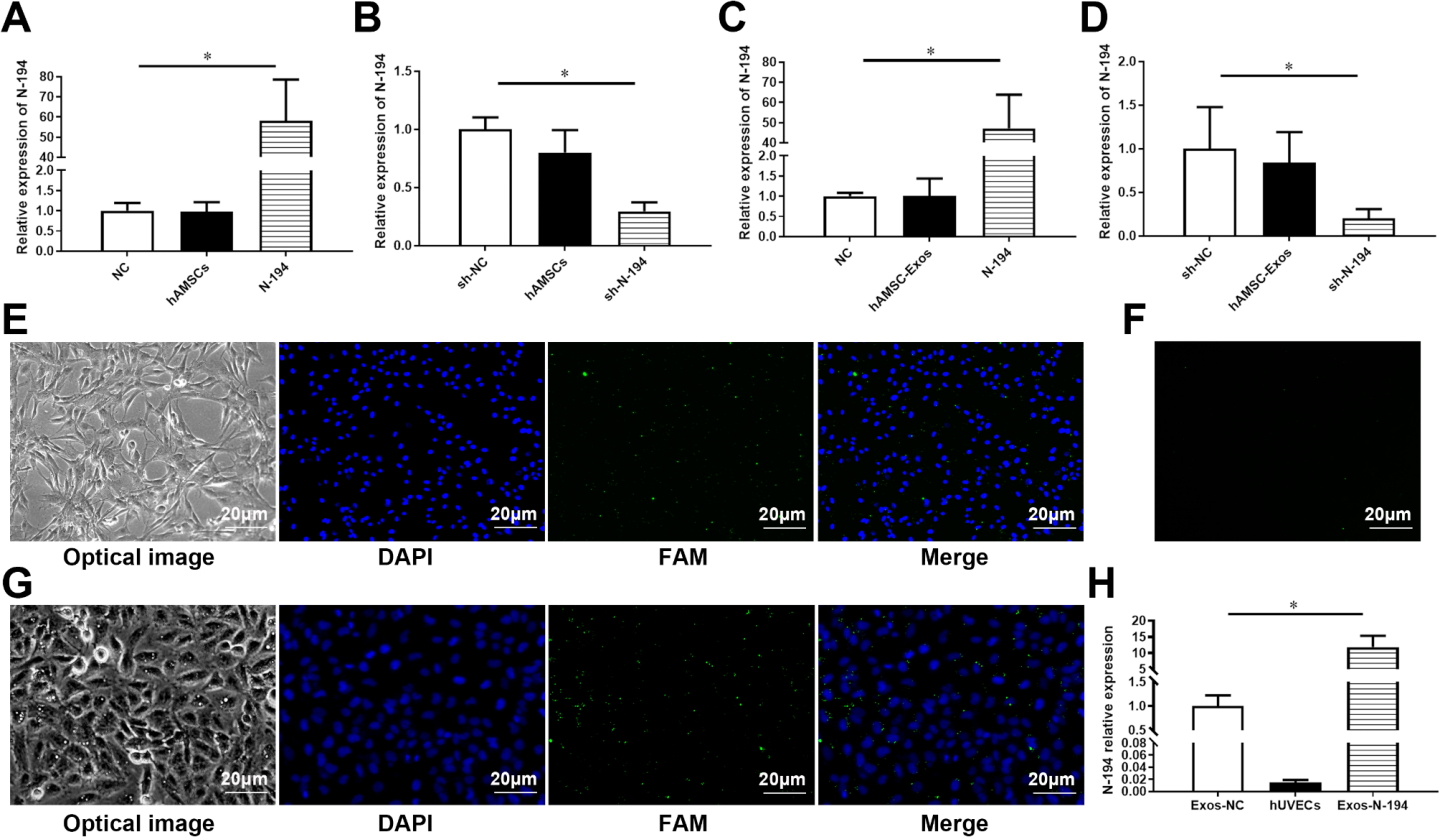
HAMSC-Exos deliver transfected N-194 to hUVECs. (**A**) After transfection of N-194 mimics and NC into hAMSCs, RT-qPCR was used to detect the cell transfection efficiency. (**B**) After transfection of sh-N-194 and sh-NC into hAMSCs, RT-qPCR was used to detect the transfection efficiency. (**C**) RT-qPCR was used to detect the expression of N-194 in hAMSC-Exos isolated from N-194 mimics and NC transfected hAMSCs. (**D**) RT-qPCR was used to detect the expression of N-194 in hAMSC-Exos isolated from sh-N-194 and sh-NC transfected hAMSCs. (**E**) FAM-labeled miRNA N-194 mimics had been transfected into hAMSCs. DAPI stained hAMSC nuclei. Scale bar = 20 μm. (**F**) FAM-labeled miRNA N-194 mimics had been detected in hAMSC-Exos. DAPI stained hAMSC nuclei. Scale bar = 20 μm. (**G**) HAMSC-Exos delivering transfected FAM-labeled N-194 to HUVECs. DAPI stained hAMSC nuclei. Scale bar = 20 μm. (**H**) After transfection of miRNA N-194 mimics and NC into hAMSCs, Exos were extracted and incubated with HUVECs. RT-qPCR was used to detect the expression of N-194. *n* = 3, data were presented as mean (standard) deviation. * *P* < 0.05, compared with NC or sh-NC group

In order to detect the function of N-194 in hAMSC-Exos, we transfected N-194 mimics, sh-N-194 and the corresponding NCs into hAMSCs by lipo2000 transfection reagent. RT-qPCR results verified that the expression of N-194 was significantly increased after transfection with N-194 mimics, and the expression of N-194 was significantly decreased after transfection of sh-N-194 (Fig. 4A and B). The transfection efficiency was very high, achieving [nearly 60 times or 37%] under the given conditions.

The Exos of hAMSCs overexpressing miRNA N-194 and NC group were extracted and the exosomal RNA was extracted. RT-qPCR was used to detect the relative expression of N-194 in hAMSC-Exos in blank control group, NC group and miRNA N-194 overexpression group. The results showed that the expression of N-194 in hAMSC-Exos in N-194 overexpression group was significantly higher than that in NC group (Fig. 4C). The Exos of hAMSCs in blank control group, Sh-NC group and sh-N-194 group were extracted, and the exosomal RNA was extracted. The relative expression of N-194 was detected by RT-qPCR. The results showed that the expression of N-194 in hAMSC-Exos in sh-N-194 group was significantly lower than that in Sh-NC group (Fig. 4D).

To demonstrate the delivery of N-194 which transfected in hAMSCs into target cells by hAMSC-Exos, we transfected Fluorescein (FAM)-labeled miRNA N-194 mimics into hAMSCs using lipo2000 transfection reagent. Figure 4E and F showed that FAM-labeled miRNA N-194 mimics have been transfected into hAMSCs and hAMSC-Exos. 100 µg /mL hAMSC-Exos was co-incubated with hUVECs. After 48 h, 4% paraformaldehyde was used to fix the cells, DAPI was used to stain the nucleus, and the cell state was observed under an inverted fluorescence microscope. The results showed that FAM was positively expressed in the cells (Fig. 4G), which indicated that hAMSC-Exos could deliver the transfected N-194 to hUVECs. RT-qPCR further demonstrated the results (Fig. 4H).

### HAMSC-Exos promote hUVECs viability, migration, and angiogenesis through N-194

**Fig. 5:**
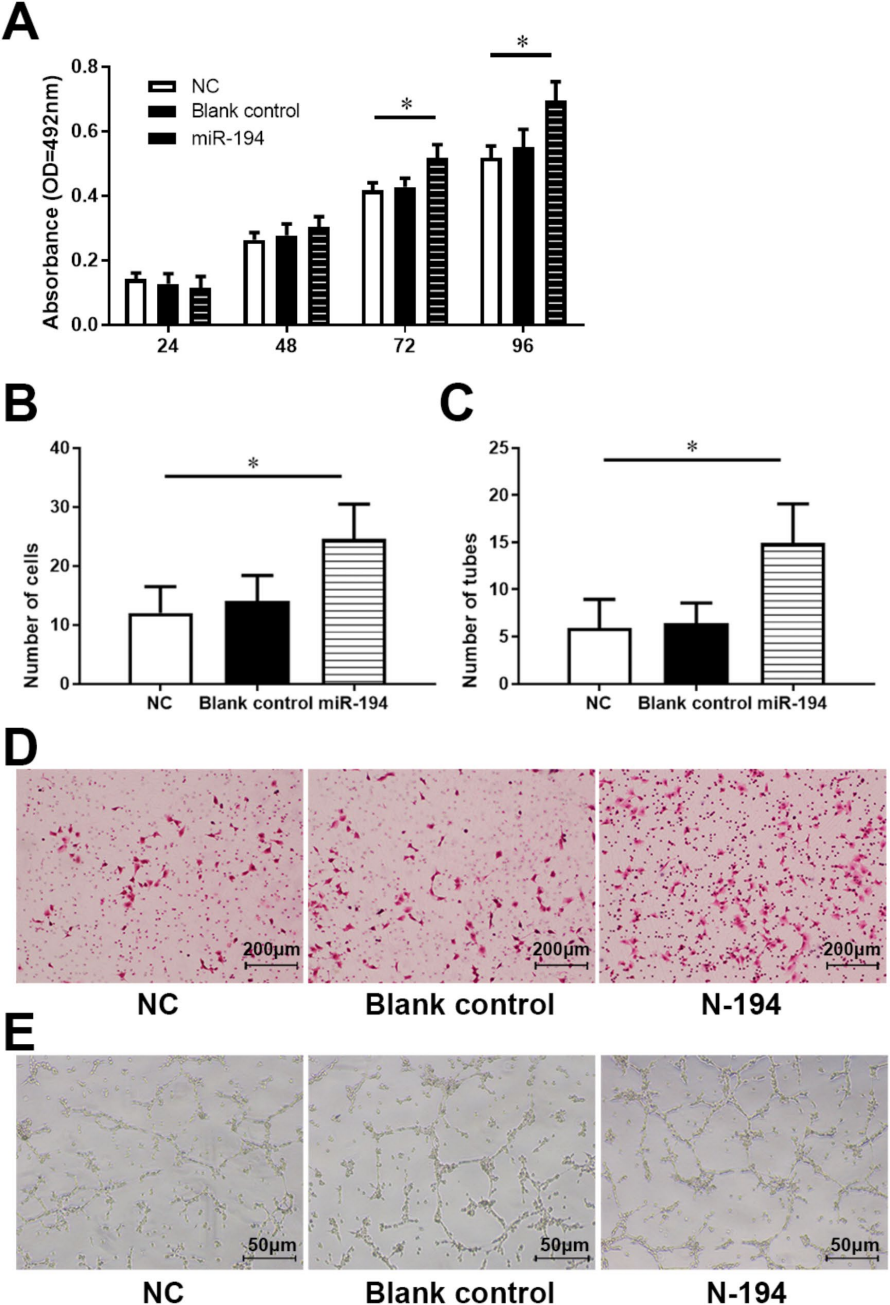
N-194 promote the viability, migration and angiogenesis of hUVECs. (**A**) Cell viability assay was used to detect the viability of hUVECs in NC group, untransfected group, N-194 transfected group. *n* = 6. (**B**) The migration ability of hUVECs in each group was detected by Transwell migration assay. *n* = 12. (**C**) Matrigel tube formation assay was used to detect the angiogenic ability of hUVECs in each group. *n* = 9. (**D**) Representative photographs of Transwell migration assay. Scale bar = 200 μm. (**E**) Representative photographs of Matrigel tube formation assay. Scale bar = 100 μm. Data were presented as mean (standard deviation). * *P* < 0.05, compared with NC group

To investigate the effect of N-194 in hUVECs, N-194 mimics and NC were transfected into hUVECs by lipo 2000 transfection reagent. Cell viability assay was used to detect the viability of hUVECs. The results showed that overexpression of N-194 significantly promotes the viability of hUVECs compared with the NC group (Fig. 5A). It was further detected that N-194 affects the migration ability of hUVECs. Transwell migration assay demonstrated that overexpression of N-194 significantly promotes the migration of hUVECs (Fig. 5B and D). Matrigel tube formation assay demonstrated that overexpression of N-194 significantly promotes the angiogenic ability of hUVECs (Fig. 5C and E). N-194 promotes viability, migration, and angiogenesis of hUVECs.

**Fig. 6:**
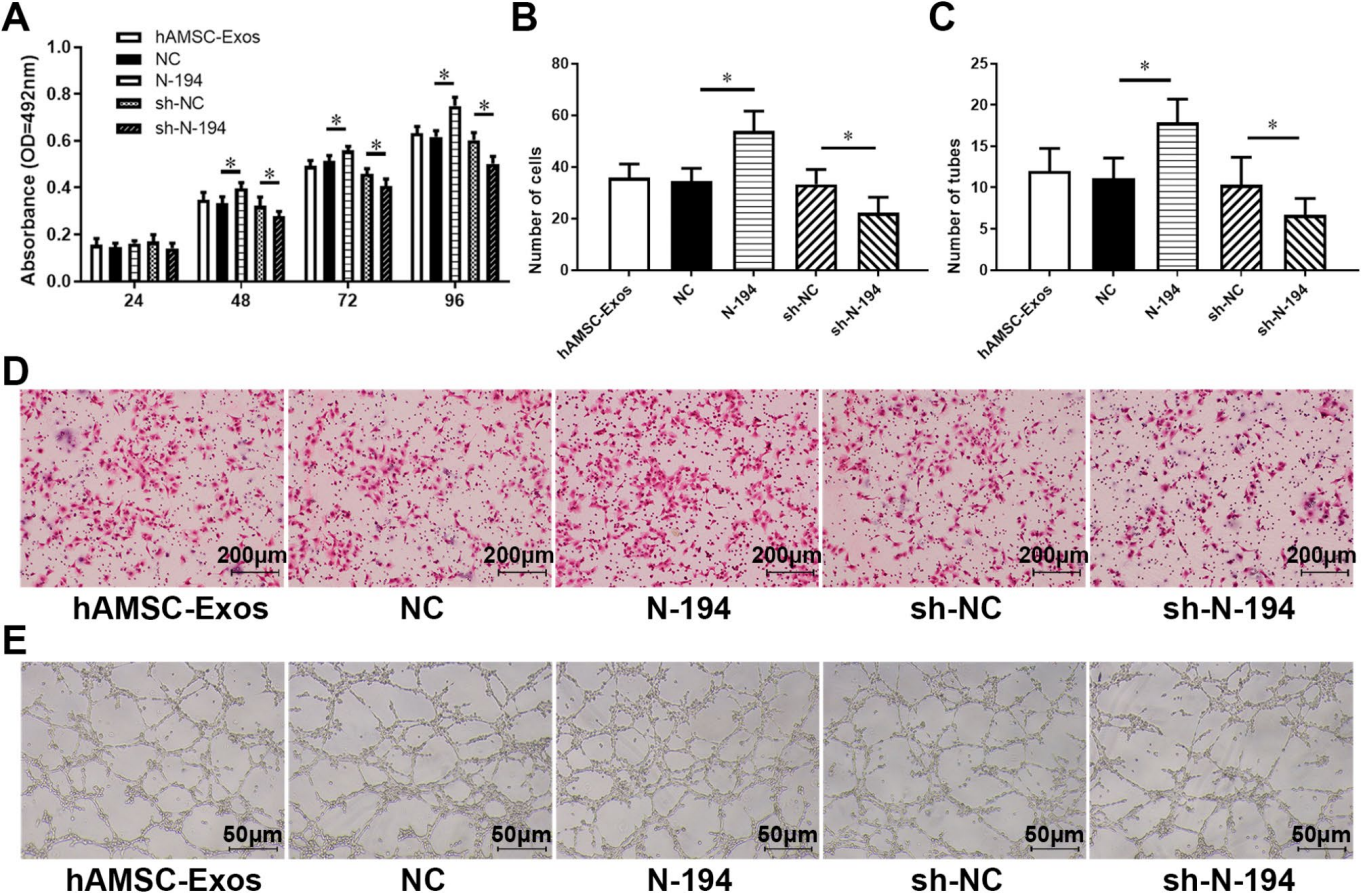
HAMSC-Exos promote the viability, migration and angiogenesis of hUVECs through N-194. (**A**) Cell viability assay was used to detect the viability of hUVECs in untransfected group, N-194 over-expression group, sh-N-194 group and respective NC groups. *n* = 6. (**B**) The migration ability of hUVECs in each group was detected by Transwell migration assay. *n* = 12. (**C**) Matrigel tube formation assay was used to detect the angiogenic ability of hUVECs in each group. *n* = 9. (**D**) Representative photographs of Transwell migration assay. Scale bar = 200 μm. (**E**) Representative photographs of Matrigel tube formation assay. Scale bar = 100 μm. Data were presented as mean (standard deviation). * *P* < 0.05, compared with NC or sh-NC group

To investigate the effect of N-194 in hAMSC-Exos on vascular regeneration, N-194 mimics, sh-N-194 and their NCs were transfected into hAMSCs by lipo 2000 transfection reagent, and hAMSC-Exos were further isolated. 100 µg/mL hAMSC-Exos were added into the medium of hUVECs and cultured in the incubator. Cell viability assay was used to detect the viability of hUVECs. The results showed that N-194 over-expression group hAMSC-Exos significantly promote the viability of hUVECs compared with the NC group, N-194 low-expression group hAMSC-Exos significantly inhibit the viability of hUVECs compared with the sh-NC group (Fig. 6A).

It was further detected that hAMSC-Exos affect the migration ability of hUVECs through N-194. Transwell migration assay demonstrated that N-194 overexpression group hAMSC-Exos significantly promoted the migration of hUVECs, N-194 low-expression group hAMSC-Exos significantly inhibit the migration of hUVECs (Fig. 6B and D).

Matrigel tube formation assay demonstrated that N-194 overexpression group hAMSC-Exos significantly promoted the angiogenic ability of hUVECs, N-194 low-expression group hAMSC-Exos significantly inhibit the angiogenic ability of hUVECs (Fig. 6C and E). HAMSC-Exos may promote viability, migration, and angiogenesis of hUVECs through delivering N-194.

### ING5 is a target gene of N-194

**Fig. 7:**
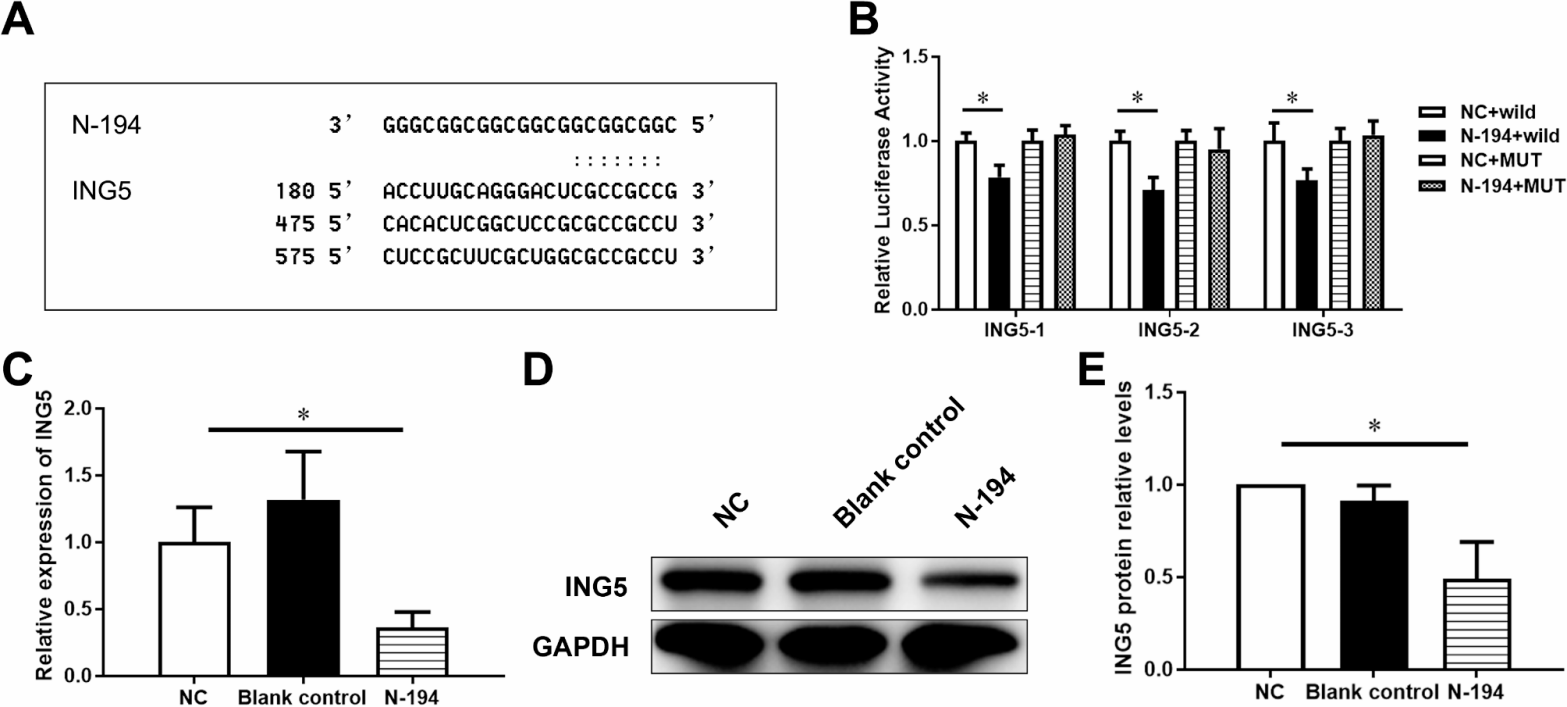
ING5 is a target gene of N-194. (**A**) TargetScan human 8.0 predicts that N-194 may regulate ING5. (**B**) Dual luciferase reporter assay was used to analyze the effect of N-194 on the expression of pGL3-ING5-1-3’UTR, pGL3-ING5-2-3’UTR and pGL3-ING5-3-3’UTR luciferase reporter genes in hUVECs. *n* = 6, compared with NC + wild group. (**C**) By lipo 2000 transfection reagent, we transfected N-194 mimics into hUVECs, and further detected the expression of ING5 in cells by by RT-qPCR. *n* = 3. (**D**) By lipo 2000 transfection reagent, we transfected N-194 mimics into hUVECs, and further detected the expression of ING5 in cells by Western blot. (**E**) The results of Western blot were scanned and statistically analyzed. *n* = 3, data were presented as mean (standard deviation). * *P* < 0.05, compared with NC group

To explore the mechanism of angiogenesis promoted by N-194 in hAMSC-Exos, TargetScan Human 8.0 was used to predict the target genes of N-194. Inhibitor of growth 5 (ING5) was demonstrated to be a potential target gene of N-194 (Fig. 7A).

In order to verify the targeted binding relationship between N-194 and ING5, we constructed wild-type and mutant DNA sequences of three DNA sequences around the predicted binding site of ING5, and combined the amplified DNA sequences with pGL3-control vector to construct the corresponding wild-type and mutant firefly luciferase reporter vectors, respectively. Firefly luciferase reporter gene, Renilla luciferase reporter gene and miRNA N-194 mimics or NC were cotransfected into hUVECs for dual luciferase reporter assay. The experimental results showed that N-194 significantly inhibit the luciferase reporter activity of three reporter genes pGL3-ING5-1-3’UTR, pGL3-ING5-2-3’UTR and pGL3-ING5-3-3’UTR (Fig. 7B).

To further detect the regulatory ability of N-194 on ING5 in hUVECs, we transfected N-194 mimics and NC into hUVECs, and detected the mRNA level and protein expression of ING5 in cells by RT-qPCR and Western blot. The results showed that N-194 significantly reduce the expression of ING5 in hUVECs (Fig. 7C-E). These demonstrated that ING5 is a target gene of N-194.

### N-194 promotes angiogenesis of hUVECs by regulating ING5

**Fig. 8:**
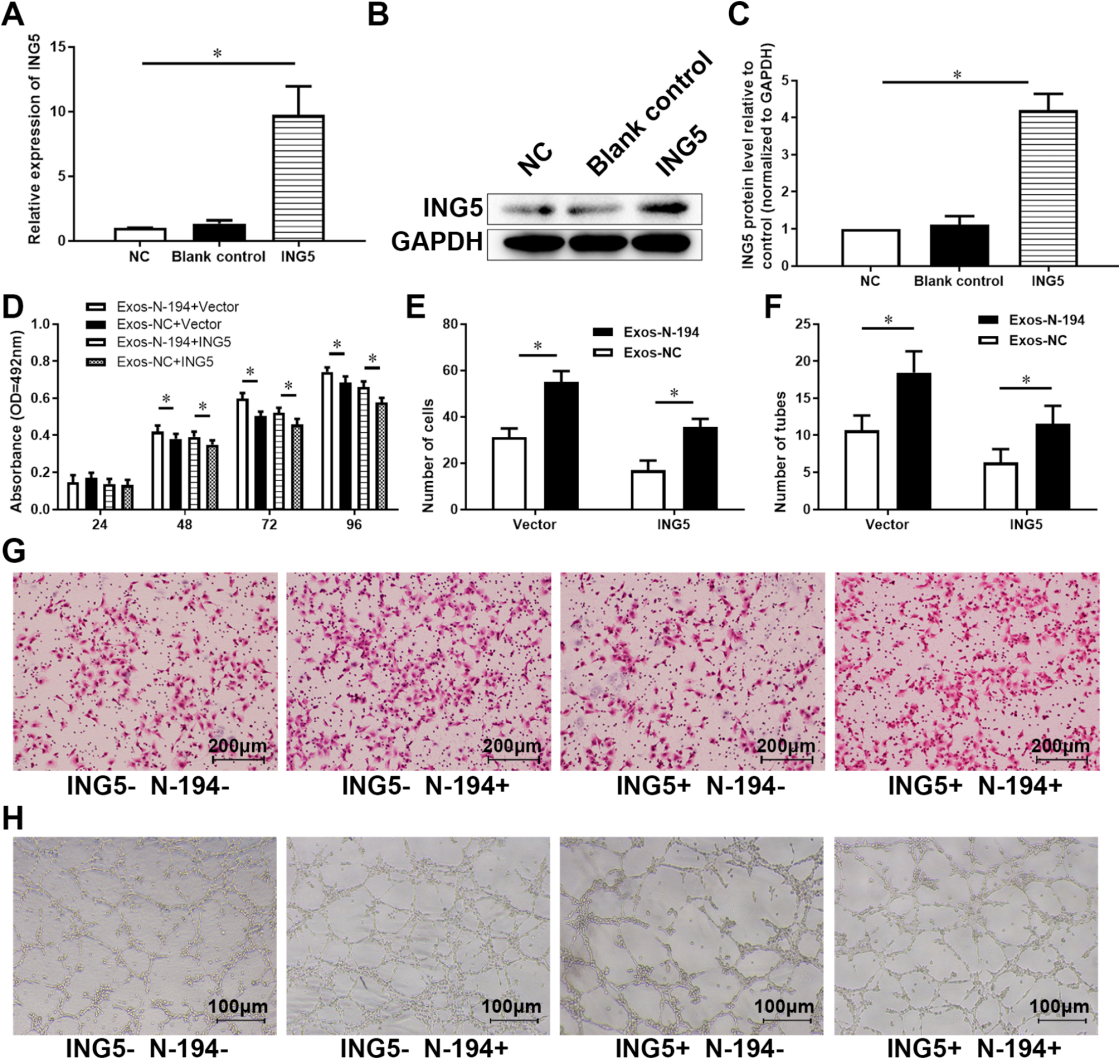
N-194 promotes the viability, migration and angiogenesis of hUVECs by inhibiting ING5. (**A**) The ING5 expression vector was transfected into hUVECs by lipofection. After 48 h, the mRNA level of ING5 in cells was detected by RT-qPCR. (**B**) Western blot was used to detect the expression of ING5 in cells. (**C**) Gray scale analysis and statistics of Western blot results. *n* = 3. (**D**) HAMSC-Exos of N-194 over-expression group and NC group were added to hUVECs transfected with ING5 expression vector and control group vector, respectively. Cell viability assay was used to detect the viability of hUVECs. *n* = 6. (**E**) Transwell migration assay was used to detect the migration ability of hUVECs. The results were statistically analyzed. *n* = 12. (**F**) The angiogenic ability of hUVECs was detected by Matrigel tube formation assay. *n* = 9. (**G**) Representative photographs of Transwell migration assay, scale bar = 200 μm. (**H**) Representative photographs of Matrigel tube formation assay, scale bar = 100 μm. Data were presented as mean (standard deviation). * *P* < 0.05, compared with the control group

To clarify that ING5 is the target of N-194 which promotes angiogenesis of hUVECs, we transfected the ING5 vector into hUVECs by lipo 2000 transfection reagent and verified the transfection efficiency (Fig. 8A-C).

The role of ING5 in the angiogenesis of hUVECs promoted by N-194 was demonstrated. HAMSC-Exos of N-194 over-expression group and NC group were added to hUVECs cells transfected with ING5 expression vector and control group vector, respectively. After 24 h, cell viability assay, Transwell migration assay and Matrigel tube formation assay showed that hAMSC-Exos overexpressed with N-194 promoted the viability, migration and angiogenesis of hUVECs by inhibiting the expression of ING5 in hUVECs (Fig. 8D-H).

**Fig. 9:**
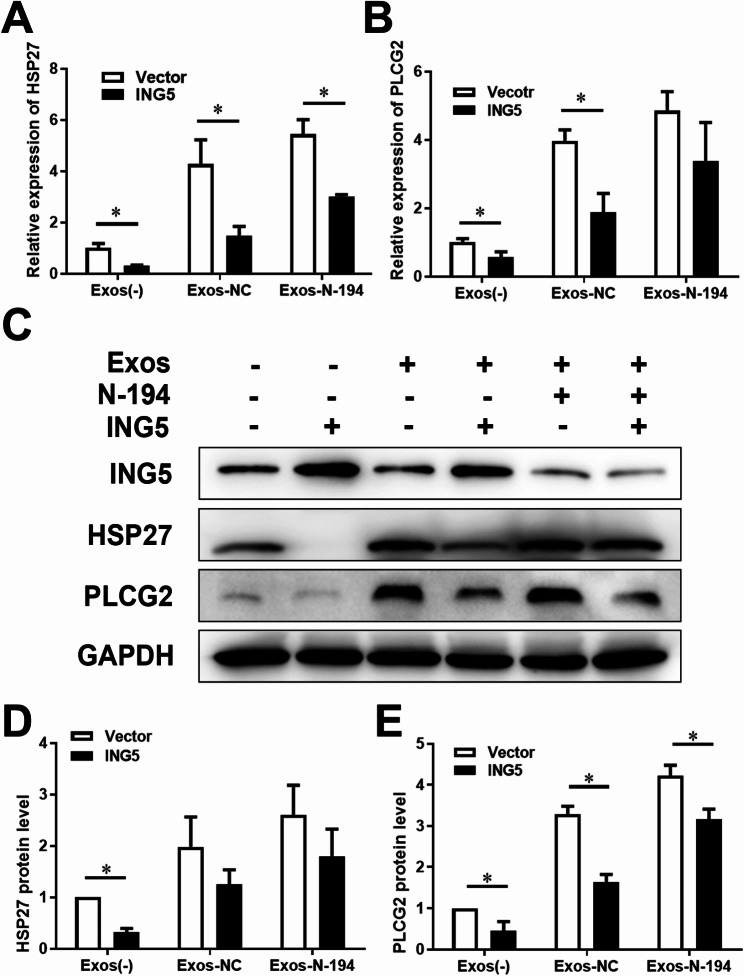
N-194 affects mRNA and protein levels of HSP27 and PLCG2 by regulating ING5. HAMSC-Exos of N-194 over-expression group and NC group were added to hUVECs transfected with ING5 expression vector, and hUVECs without hAMSC-Exos group were used as control group for comparison. (**A**) After 24 h, the mRNA level of HSP27 in hUVECs cells was detected by RT-qPCR. (**B**) The mRNA level of PLCG2 in hUVECs cells was detected by RT-qPCR. (**C**) The expression of ING5, HSP27 and PLCG2 in hUVECs was detected by Western blot. (**D**) Analysis and statistics of HSP27 Western blot results. (**E**) Analysis and statistics of PLCG2 Western blot results. *n* = 3, data were presented as mean (standard deviation). * *P* < 0.05, compared with the vector group

To investigate the mechanism of angiogenesis promoted by hAMSC-Exos delivered N-194 which targets ING5, we detected the mRNA and protein levels of Vascular endothelial growth factor (VEGF) pathway genes heat shock protein 27 (HSP27) and phospholipase c-gamma 2 (PLCG2). RT-qPCR and Western blot showed that N-194 significantly affected the mRNA and protein levels of HSP27 and PLCG2 by regulating ING5 (Fig. 9).

## Discussion

MSCs therapy provides new hope for the treatment of diseases and may become one of the most promising therapeutic methods with the maturity of stem cell technology (Uccelli et al. 2008). Blood supply provides important nutrients for tissue regeneration (Van et al. 2021). The formation of functional tissues depends on blood supply. Through promoting vascular regeneration, MSCs significantly promote the regeneration of many tissues, such as: bone (Zigdon-Giladi et al. 2015), skin, myocardium (Watt et al. 2013), and kidney (Brasile et al. 2019). Studies have shown that MSCs play a significant role through secreting Exos in the treatment of a variety of ischemic diseases including myocardial infarction, stroke, liver ischemia, kidney ischemia, and so on (Gilbert-Honick et al. 2020; Kim et al. 2020; Sun et al. 2020; Tsuji et al. 2018).

Exos which are important parts of the extracellular vesicles secreted by MSCs play important roles of MSCs. They can deliver the young factors of MSCs to target cells by carrying components such as miRNAs, mRNA and proteins, and combine with target cells to change their state (Toh et al. 2018; Zhang et al. 2015). Studies have shown that MSCs derived Exos can promote angiogenesis, repair damaged tissues, regulate immunity, anti inflammation and inhibit apoptosis (Gao et al. 2022; Liao et al. 2021; Shi et al. 2022; Wu et al. 2018). Through previous research, we found that hAMSCs have a significant promoting effect on angiogenesis by paracrine effect (Wu et al. 2017). To investigate the effect of hAMSC-Exos on vascular endothelial cells, hAMSC-Exos were isolated by ultrafiltration and identified by transmission electron microscopy, NTA and Western blot. The viability, migration and angiogenesis of vascular endothelial cells are the basis of their functions (Eelen et al. 2018; Herbert et al. 2011; Laddha et al. 2019). In this study, we found that 50 and 100 µg/mL of hAMSC-Exos signigicantly promote the viability, migration and angiogenesis of hUVECs. This is consistent with the results of hAMSC-Exos facilitate Diabetic wound healing by angiogenesis (Fu et al. 2023).

MiRNAs are about 20 nucleotides small non coding RNAs that widely exist in various eukaryotes. These small RNAs originate from a small fragment in RNA and bind with target gene mRNA to degrade target gene mRNA or inhibit the protein expression of coding genes (Liu et al. 2014). Exos contains a large number of miRNAs, which play important regulatory roles through the delivery of miRNAs (Zhang et al. 2015). To clarify the mechanism by which hAMSC-Exos promote angiogenesis, we analyzed miRNAs expressed in hAMSC-Exos. Through the next-generation small RNA sequencing and bioinformatics analysis of hAMSC-Exos, we identified the expression of new miRNAs N-194, N-314, N-19, N-393 and N-481 in hAMSC-Exos. Moreover, the relative expression of the new miRNAs in hAMSC-Exos was detected by stem loop RT-qPCR. The results showed that the expression of N-194 was the highest.

Exos can deliver the miRNAs contained in it to target cells and play a significant role. To clarify the function of hAMSC-Exos in delivering N-194, we transfected FAM-labeled miRNA N-194 mimics into hAMSCs using lipo2000 transfection reagent, and co-incubated the extracted hAMSC-Exos with hUVECs. The results showed that hAMSC-Exos could deliver the transfected FAM-labeled N-194 to hUVECs, which significantly increased the expression of N-194 in hUVECs. This provides a sufficient theoretical basis for hAMSC-Exos to function by delivering N-194.

In order to investigate the mechanism of hAMSC-Exos delivering N-194 to promote angiogenesis, we transfected N-194 mimics, sh-N-194 and the corresponding NCs into hAMSCs by using lipo2000 transfection reagent, and isolated hAMSC-Exos from N-194 over-expression group, N-194 low-expression group and two NC groups by ultracentrifugation. Furthermore, hAMSC-Exos differentially expressing N-194 was further incubated with hUVECs. Cell viability assay, Transwell migration assay and Matrigel tube formation assay showed that hAMSC-Exos over-expressing N-194 could significantly promote the viability, migration, and angiogenic ability of hUVECs, while hAMSC-Exos with low-expression of N-194 had significantly reduced the ability to promote the viability, migration, and angiogenesis of hUVECs. This indicates that N-194 is an important molecular mechanism for hAMSC-Exos to promote angiogenesis, and hAMSC-Exos delivers N-194 to vascular endothelial cells to promote angiogenesis. Exos have strong homing capacity (Araldi et al. 2023). N-194 can be used as a pro-angiogenic factor which Exos affect target cells.

To clarify the molecular mechanism of N-194 in hAMSC-Exos promoting angiogenesis of hUVECs. We used TargetScan human 8.0 and dual luciferase reporter assay to prove that ING5 is a target gene of N-194. RT-qPCR and Western blot demonstarted that the mRNA and protein expression of ING5 decreases to below 50% after transfected with N-194. The growth inhibitor (ING) family consists of ING1, ING2, ING3, ING4, and ING5 (Dantas et al. 2019). ING regulates cell viability, senescence, apoptosis, differentiation, migration and angiogenesis through a variety of pathways (Dantas et al. 2019; Shi et al. 2005). ING family is significantly correlated with angiogenesis. ING1 affects the expression of HSP70 and may therefore be involved in the process of angiogenesis (Feng et al. 2006). Nuclear localization of ING3 is required to suppress melanoma cell angiogenesis. (Zhou et al. 2020). Knockout of ING4 strongly affects angiogenesis (Dantas et al. 2019). ING5 is involved in many important cellular functions (Ludwig et al. 2011; Zhang et al. 2017). However, the role of ING5 in angiogenesis is still unclear. In order to clarify that ING5 is the target of N-194 promoting the angiogenesis of hUVECs, we demonstrated the role of ING5 in the process of N-194 promoting the angiogenesis of hUVECs. Our research provides more evidence that ING family play important roles in angiogenesis.

Angiogenesis which is essential for normal development, tissue homeostasis and organ repair is regulated by the activation of angiogenic signals induced by angiogenic factors. VEGFA, a key regulator of angiogenesis, binds to VEGF receptor 2 (VEGFR2) during the early process of VEGFA signaling, leading to VEGFR2 phosphorylation and internalization through endocytic vesicles, which in turn activates phospholipase c-gamma (PLCG) transmits signals downward to turn on the angiogenesis process (Watari et al. 2020). As an important member of PLCG, PLCG2 encodes a transmembrane signaling enzyme that functions downstream of VEGF signaling. This signaling molecule is required for hematopoietic cell differentiation and function (Rustagi et al. 2022). Inhibition of PLCG2 can significantly inhibit VEGF signaling pathway, thereby blocking the process of angiogenesis. We proved that N-194 regulated the expression level of PLCG2 by regulating ING5, and then promoted angiogenesis.

HSP27 is a small, ATP independent concomitant molecule induced under cellular stress conditions such as oxidative stress and heat shock, which protects proteins from unfolding, thereby promoting protein stability and cell survival (Lampros et al. 2022; Shan et al. 2021). HSP27 is also an important factor downstream of VEGF signaling pathway. Studies have shown that HSP27 also plays an important role in the process of VEGF induced angiogenesis (Lampros et al. 2022). HSP27 can interact with Toll-like receptor 3 (TLR3) to induce nuclear factor-kappa B (NF-κB) pathway activation, causing VEGF mediated angiogenesis (Thuringer et al. 2013). Downregulation of HSP27 expression inhibited VEGF induced membrane protrusion and migration, which in turn inhibited angiogenesis (Sawada et al. 2015). Through experiments, we proved that N-194 can regulate the expression level of HSP27 by acting on ING5, and then promote angiogenesis. However, the regulatory effect of Exos-N-194 on PLCG2 and HSP27 seems weak. We thought that although the N-194 expression of Exos-N-194 was significantly higher, there are so many target genes of N-194. So, the effect of N-194 overexpression on ING5 expression was not that strong. Besides, the effect of Exos-N-194 regulating HSP27 and PLCG2 was via regulating ING5. Perhaps the regulatory effect has weakened in this process.

## Conclusion

HAMSC-Exos promote angiogenesis in hUVECs by delivering novel miRNA N-194 which targets ING5.

## Electronic supplementary material

Below is the link to the electronic supplementary material.

Supplementary Material 1

Supplementary Material 2

## Data Availability

All data including Sanger sequencing data supporting the conclusions of this article are available from the corresponding author upon request.

## Abbreviations

hAMSC-Exos: Human Amniotic Mesenchymal Stem Cells
MSCs: Mesenchymal Stem Cells
Exos: Exosomes
hAMSCs: Human Amniotic Mesenchymal Stem Cells
miRNAs: microRNAs
ING5: Inhibitor of Growth 5
VEGFR2: VEGF Receptor 2
PLCG: Phospholipase C-Gamma
HSP27: Heat Shock Protein 27
VEGF: Vascular Endothelial Growth Factor
TLR3: Toll-Like Receptor 3
NF-κB: Nuclear Factor-Kappa B
SPF: Specific Pathogen-Free
NTA: Nanoparticle Tracking Analysis
HE: Hematoxylin and Eosin
NC: Negative Control
