# Supplementary material for: Exosomes derived from human amniotic mesenchymal stem cells promotes angiogenesis in hUVECs by delivering novel miRNA N-194
Source: Mol Med. 2025 May 6;31:173. doi: 10.1186/s10020-025-01192-8 (PMC12054200; doi:10.1186/s10020-025-01192-8)

Figure 1C

CD9 (23KD)

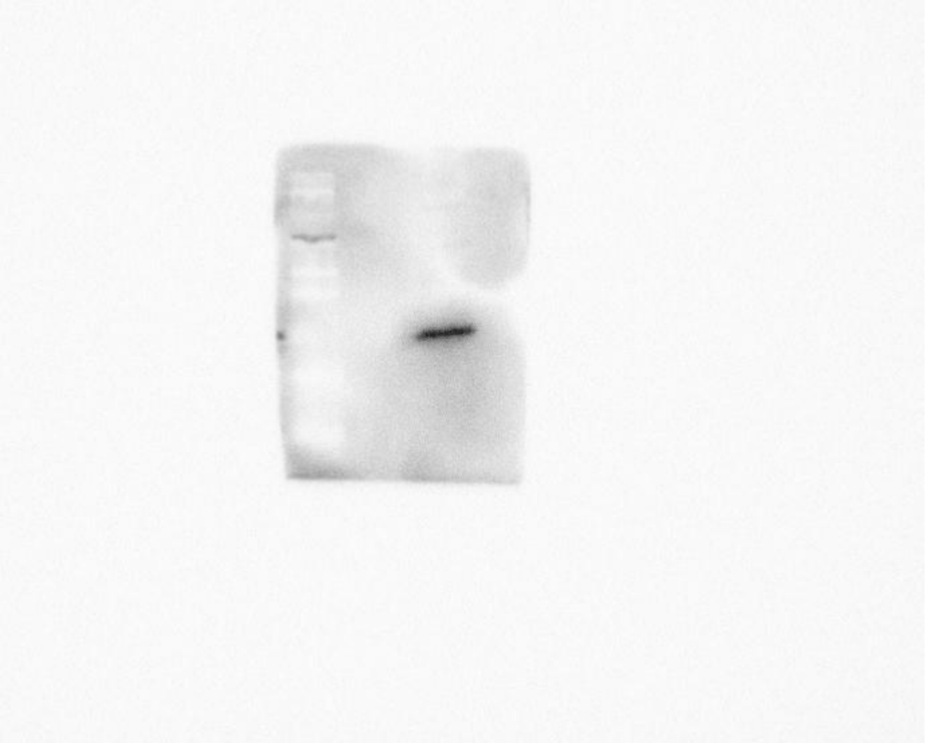

CD81 (22KD)

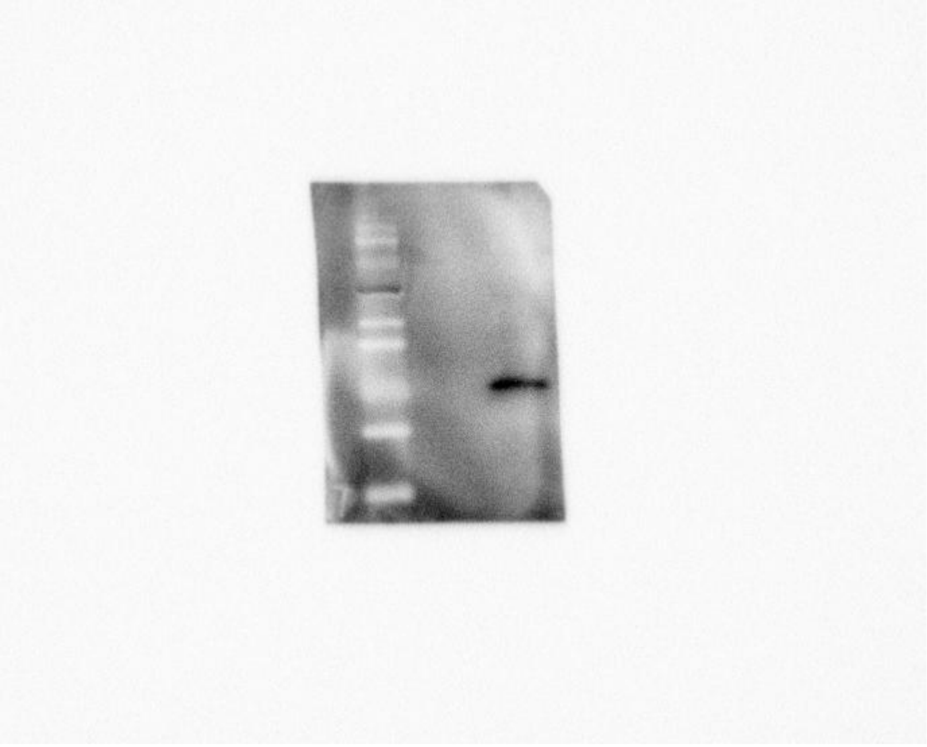

CD63 (26/60KD)

ING5 (32KD)

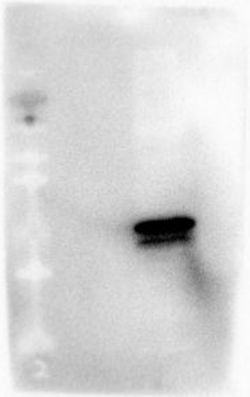

Figure 6D

GAPDH (36KD)

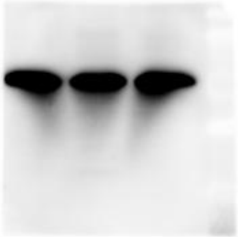

ING5 (32KD)

ING5 (32KD)

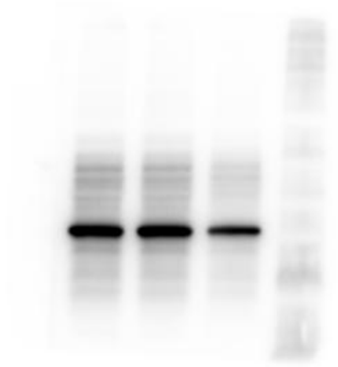

Figure 7B

GAPDH (36KD)

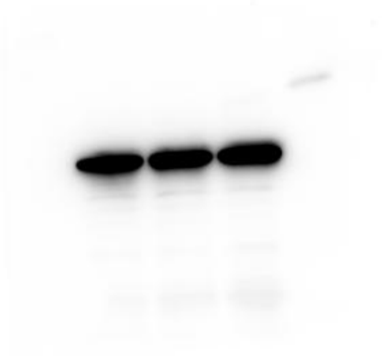

ING5 (32KD)

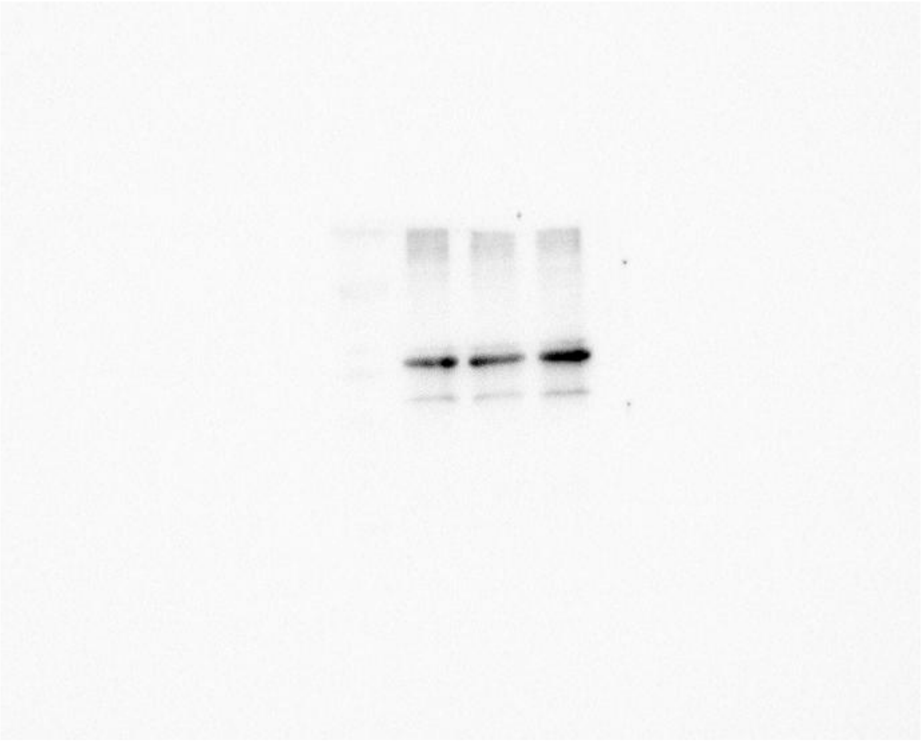

Figure 8C

HSP27 (27KD)

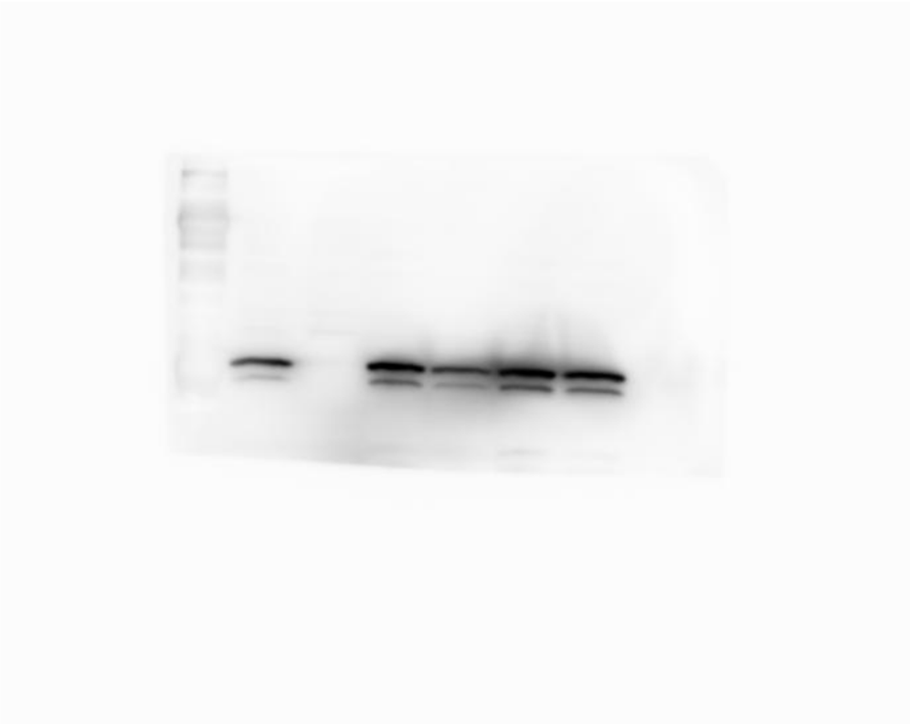

PLCG2 (148KD)

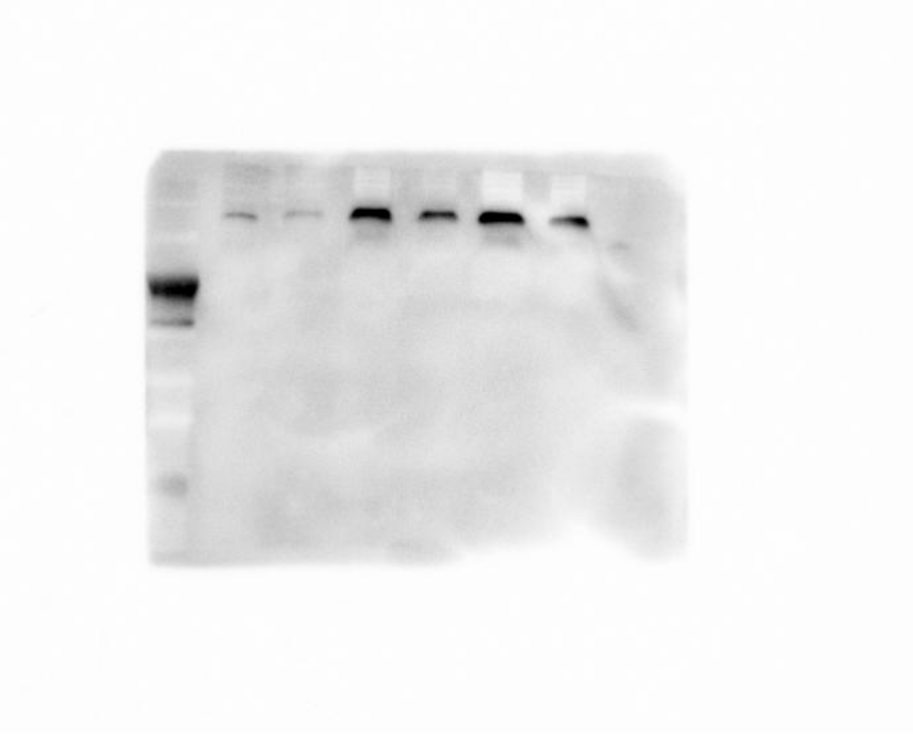

GAPDH (36KD)

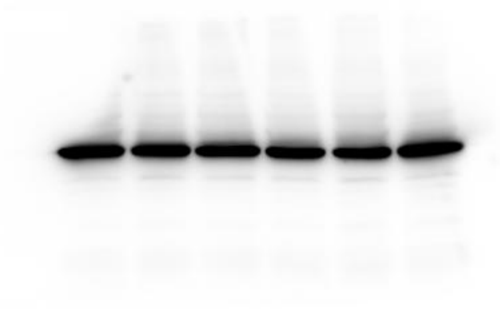

ING5 (32KD)

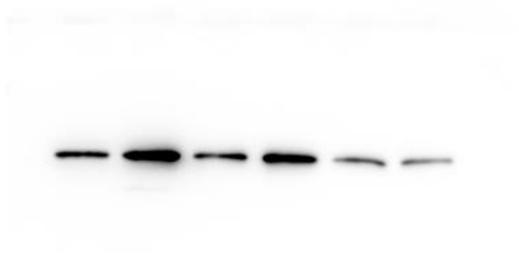

Supplement: Supplementary file 1 — Supplementary Material 1 [file 10020_2025_1192_MOESM1_ESM.pdf]
