# Supplementary material for: Exosomes derived from human amniotic mesenchymal stem cells promotes angiogenesis in hUVECs by delivering novel miRNA N-194
Source: Mol Med. 2025 May 6;31:173. doi: 10.1186/s10020-025-01192-8 (PMC12054200; doi:10.1186/s10020-025-01192-8)
Supplement: Supplementary file 2 — Supplementary Material 2 [file 10020_2025_1192_MOESM2_ESM.docx]

**Supplementary table 1. RT-qPCR primer sequences**

|  | Primer sequence (5’-3’) |
| --- | --- |
| N-194-RT | GCGTCTCAACTGGTGTCGTGGAGTCGGCAATTCAGTTGAGACGCAAAGCCGC |
| N-194 | CGGCGGCGGCGGCGGCGGCGGG |
| N-314-RT | GCGTCTCAACTGGTGTCGTGGAGTCGGCAATTCAGTTGAGACGCCGCCGCCG |
| N-314 | CGGCGGUGGCGGCGGCGGCGGCG |
| N-19-RT | GCGTCTCAACTGGTGTCGTGGAGTCGGCAATTCAGTTGAGACGCAAGCCGCC |
| N-19 | CGGCGGCGGCGGCGGCGGCGG |
| N-393-RT | GCGTCTCAACTGGTGTCGTGGAGTCGGCAATTCAGTTGAGACGCAGGGTCAG |
| N-393 | TCAAATCCTGTCTGACCCT |
| N-481-RT | GCGTCTCAACTGGTGTCGTGGAGTCGGCAATTCAGTTGAGACGCCACCGCCG |
| N-481 | CGGCGGCGGCGGCGGCGGTG |
| Universal primers | GCGTCTCAACTGGTGTCGTG |

RT：stem loop RT-qPCR primer

**Supplementary table 2. RT-qPCR primer sequences**

|  | Forward primer (5’-3’) | Reverse primer (5’-3’) | Reference |
| --- | --- | --- | --- |
| ING5 | GAGAGCTGGACCAGAGGACG | GAATGTGTTTATCCACCATCTCG |  |
| HSP27 | GTCCCTGGATGTCAACCACT | CTTTACTTGGCGGCAGTCTC | 90 |
| PLCG2 | CTGCAAACCAACCAGCAAAAC | TCAGCCTTCGTCTCCACAAA | 91 |
| GAPDH | GGAGCGAGATCCCTCCAAAAT | GGCTGTTGTCATACTTCTCATGG | 55 |

**Supplementary table 3. primer sequences for**

|  | Primer sequences / DNA sequences (5’-3’) |
| --- | --- |
| CARD11-Forward | GCTCTAGACCTGGCCTAGCAGTTT |
| CARD11-Reverse | GCTCTAGAGGGATGTGTTTATGGTG |
| ING5-1-Forward | GCTCTAGATAATCTGTCCCTTCATTC |
| ING5-1-Reverse | GCTCTAGAAAACCACAGGAAAGCT |
| ING5-2-Forward | GCTCTAGACATGGTAACCTGGTCC |
| ING5-2-Reverse | GCTCTAGATTCCTCCAGGGAAGAG |
| ING5-3-Forward | GCTCTAGACTCTTCCCTGGAGGAAG |
| ING5-3-Reverse | GCTCTAGAAGAAACGGAGCATCAG |
| CARD11-MUT | GCTCTAGACAGGGGAGCAAAGAACGCCAAGCCGGAGGCCCGAGGCCAGCCGGCCTCTCGAGAGCCAGAGCAGCAGTTGAATGTAATGCTGGGGACAGGCATAGCTATTAAAGTAGGGCGGGGACCCGGACAGCCAGGTGACTACCAGTCCTGGGGACACACTCACCATAAACACATCCCCAGGCAGGACAGATCGGGGAAGGGGTGTTCTAGAGC |
| ING5-1-MUT | GCTCTAGAACTCCGTGGCCAGTTGAAGCGCTGGATGTTTCCTAGAACAAGAACCACCAAAGCCTGTTCGCACAGAAGGGCGACCTTGCAGGGACTACGTAGTGCGACCTCAGTGTGGCTTTTACAGGACTCCCCCCGAGCATCAGCAGGGACCCCGGCGGACGTGGGCGGGCGCGCGTGAGCTCGGGCTGCCCGGCCGGGCGTGTCTAGAGC |
| ING5-2-MUT | GCTCTAGAGCTCCGCGTGCCCCGCCCGCTGGAGCACCTGCCACCGAGGCGCGCGTGGGGCCACTGCCGTGGCGGCGGCTGCCCTCCTCACACTCGGCTCCGACGTCTCTCCGGCCACCGTGCGCTCCCGCGTGGGGCGCCTCGGATGGGCCCGGGAGGGCTGGGGGCTCTTCCCTGGAGGAAGCGGCCTCCGCTTCGCTGGTCTAGAGC |
| ING5-3-MUT | GCTCTAGATCCGGCCACCGTGCGCTCCCGCGTGGGGCGCCTCGGATGGGCCCGGGAGGGCTGGGGGCTCTTCCCTGGAGGAAGCGGCCTCCGCTTCGCTGGCGCCGCCTTTTTAGCTTGGACTTCAGTCCTCCCTCGGGGACTCACCTCCGGAGTAAACGGCTCTTCATTAGCTTGGAGTGGCCGCAGGTCCCGTGACCAGCACCCGTCTAGAGC |


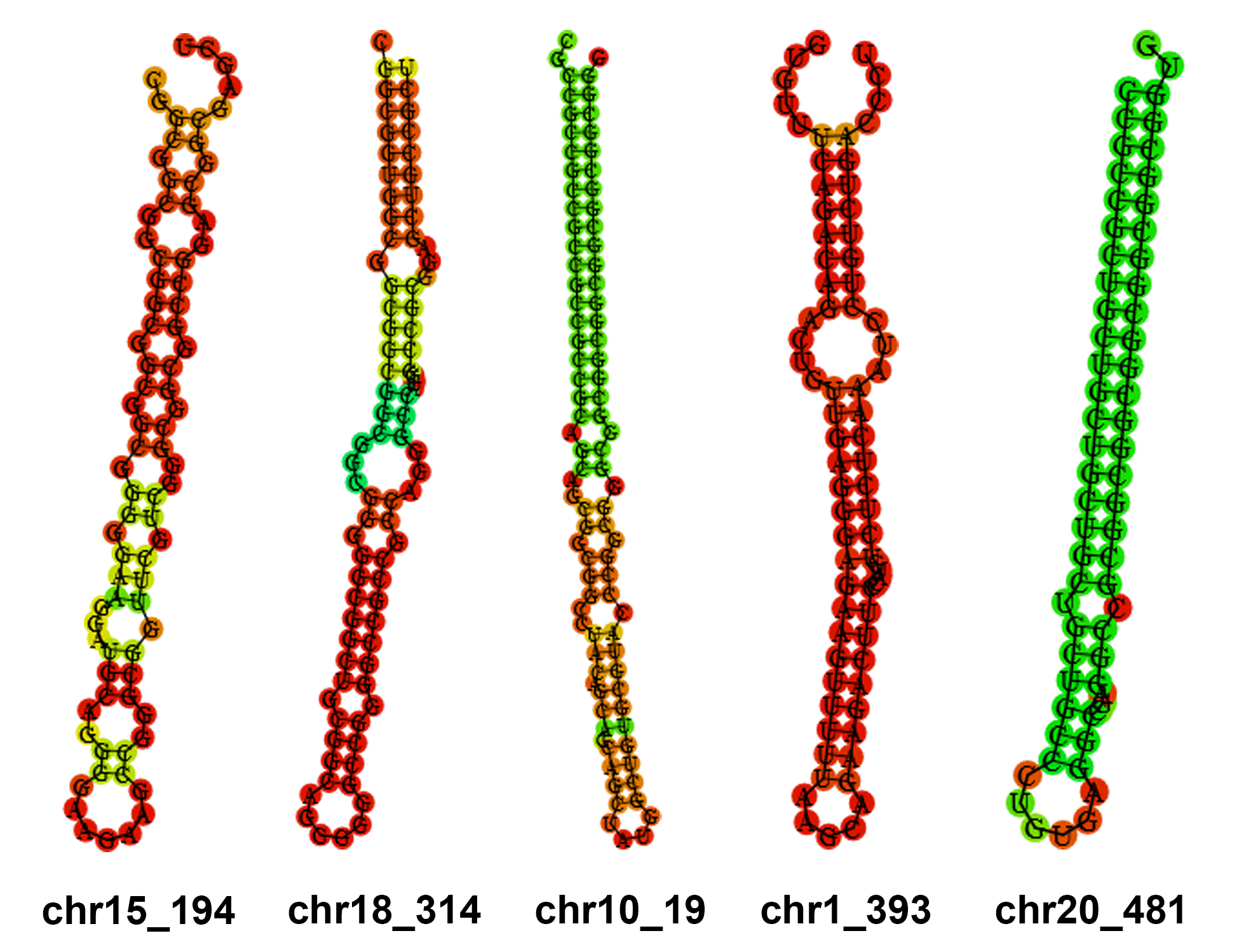


**Supplementary figure 1. Secondary structure of precursors of new miRNAs**

The secondary structures of the precursors of new miRNAs N-194, N-314, N-19, N-393, and N-481 predicted using miRDeep 2.0.


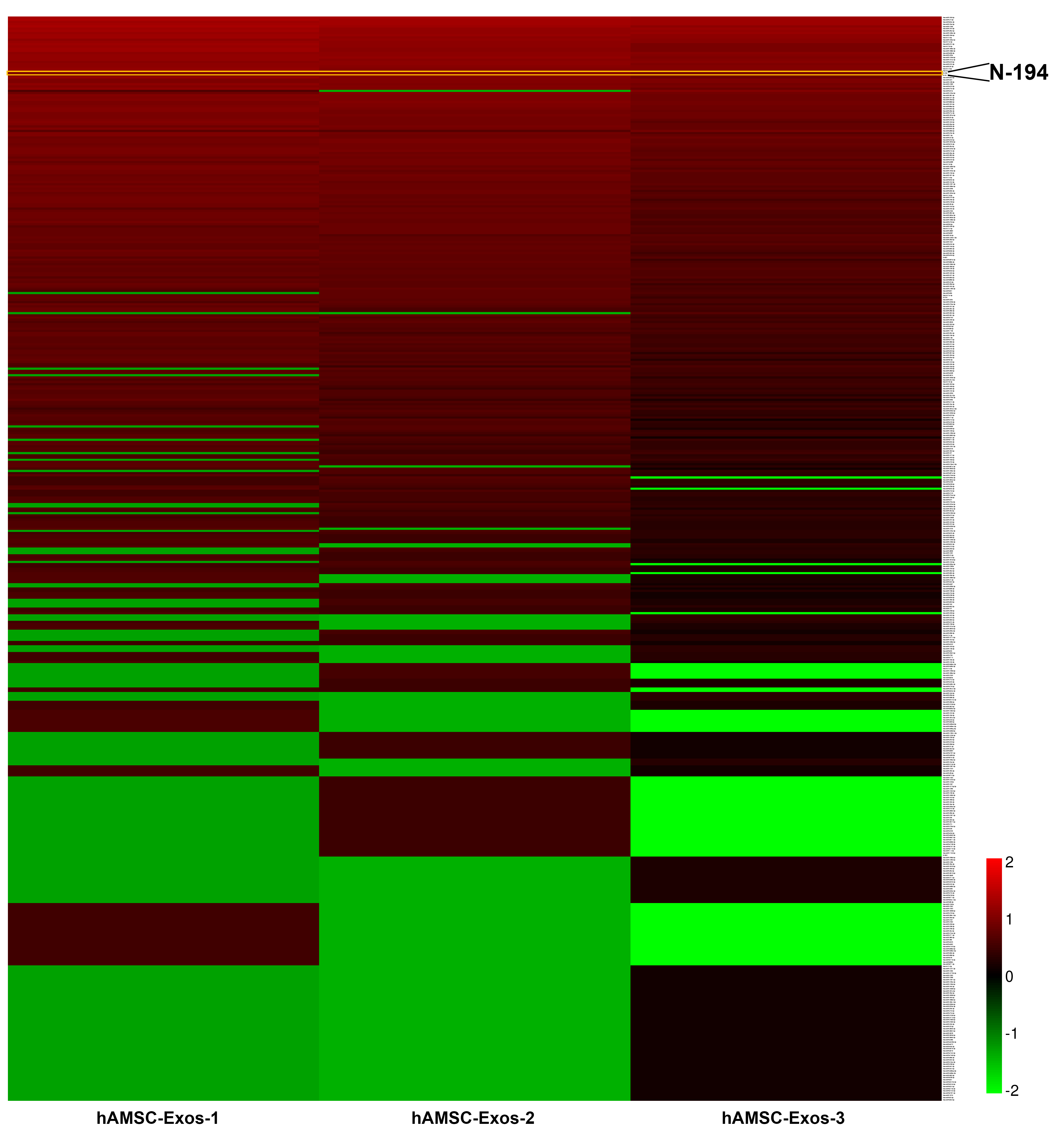


**Supplementary figure 2. Heatmap depicted the expression of miRNAs detected by next-generation small RNA sequencing of hAMSC-Exos**

Heatmap showed The expression of N-194 was very high in the hAMSC-Exos.


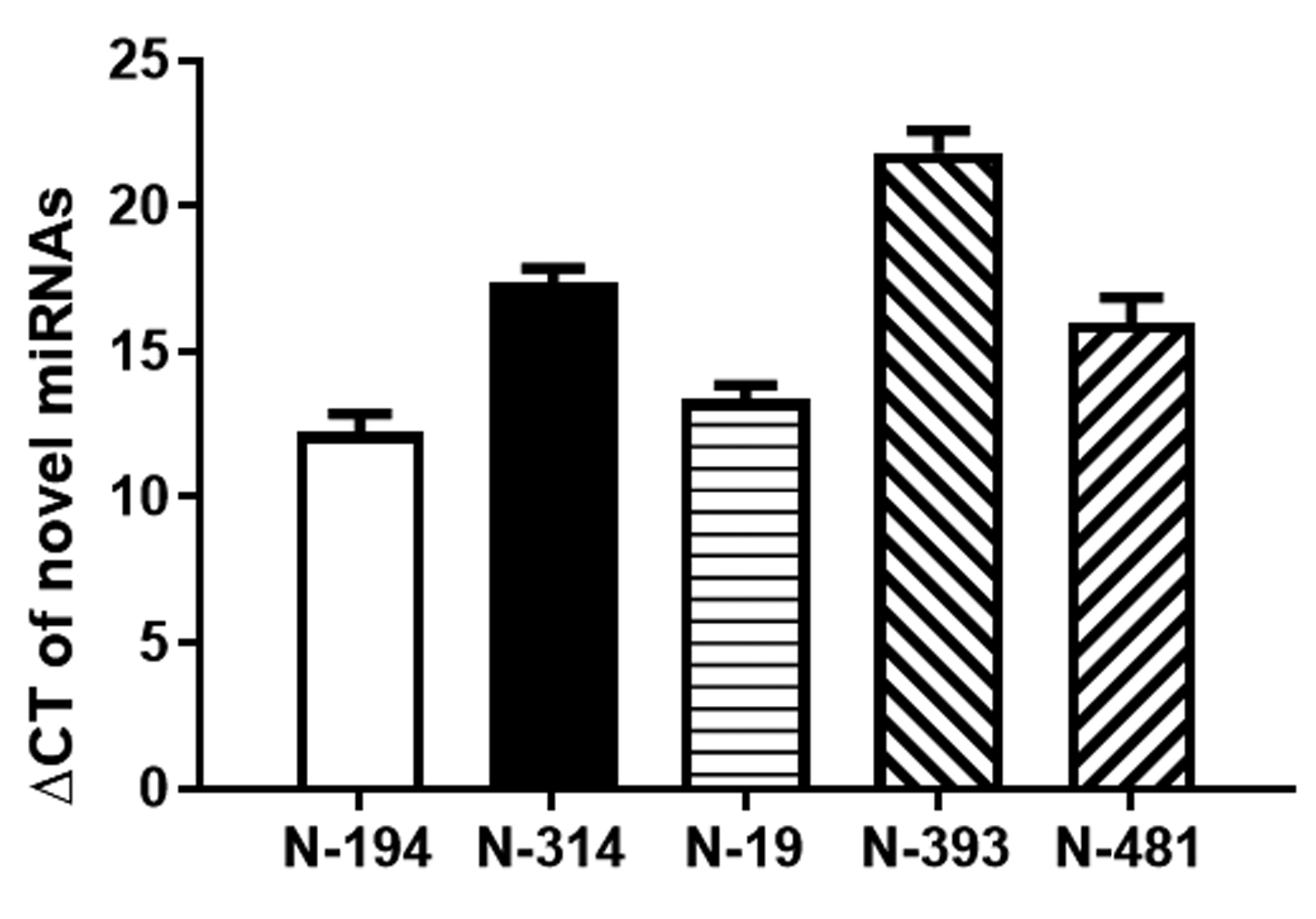


**Supplementary figure 3. Levels of new miRNAs in hAMSC-Exos**

The relative expression levels of new miRNAs N-194, N-314, N-19, N-393, and N-481 in hAMSC-Exos were detected using stem loop RT-qPCR. n=3, data were presented as mean (standard deviation).
